# Synthesis of Indole-Coupled KYNA Derivatives via C–N Bond Cleavage of Mannich Bases

**DOI:** 10.3390/ijms23137152

**Published:** 2022-06-28

**Authors:** Bálint Lőrinczi, Péter Simon, István Szatmári

**Affiliations:** 1 Institute of Pharmaceutical Chemistry, University of Szeged, Eötvös u. 6, H-6720 Szeged, Hungary; lorinczi.balint@szte.hu (B.L.); petersimon020@gmail.com (P.S.); 2 Institute of Pharmaceutical Chemistry, Interdisciplinary Excellence Center, University of Szeged, H-6720 Szeged, Hungary; 3 MTA-SZTE Stereochemistry Research Group, Hungarian Academy of Sciences, Eötvös u. 6, H-6720 Szeged, Hungary

**Keywords:** kynurenic acid, indole, Mannich base, Mannich reaction, triarylmethanes, bioconjugates

## Abstract

KYNAs, a compound with endogenous neuroprotective functions and an indole that is a building block of many biologically active compounds, such as a variety of neurotransmitters, are reacted in a transformation building upon Mannich bases. The reaction yields triarylmethane derivatives containing two biologically potent skeletons, and it may contribute to the synthesis of new, specialised neuroprotective compounds. The synthesis has been investigated via two procedures and the results were compared to those of previous studies. A possible alternative reaction route through acid catalysis has been established.

## 1. Introduction

Indole derivatives are widely distributed in nature and many of them display important biological activities; moreover, a vast number of natural and synthetic indoles have found applications as pharmaceuticals [1,2,3]. Therefore, the synthesis [4,5] and functionalization [6,7] of indoles have become hot topics of organic synthesis in recent decades [8,9] with a novel emphasis on bisindole derivatives. These compounds, be it symmetrical [10,11,12,13] or unsymmetrical compounds [14,15,16,17], have highly promising biological activities such as antitumor, arylhydrocarbon receptor modifying, and AChE inhibitor activity.

Because of their biological potential, DIMs have received much attention in organic synthesis, and several highly efficient methods have been developed for their production [18,19,20,21,22].

Another compound with biological relevance is kynurenic acid (KYNA). Among the important features of KYNA, it is known to be one of the few known endogenous excitatory amino acid receptor blockers with a broad spectrum of antagonistic properties in supraphysiological concentrations. It is well established that KYNA has high affinity toward *N*-methyl-D-aspartate (NMDA) receptors. Moreover, it has recently been disclosed that KYNA shows an even higher affinity towards the positive modulatory binding site at the α-amino-3-hydroxy-5-methyl-4-isoxazolepropionic acid (AMPA) receptor [23].

Since KYNA is a neuroprotective agent that is able to prevent neuronal loss following excitotoxic, ischemia-induced, and infectious neuronal injuries [24,25], there has recently been increasing interest in the synthesis and pharmacological studies of KYNA derivatives. The substitutions of KYNA at positions 5–8 were achieved by starting from the corresponding aniline via the modified Conrad–Limpach method [26,27,28]. The hydroxy group at position 4 was transformed to ether [28,29,30] or amine functions [31,32,33], while the carboxylic function at position 2 was mostly modified into the corresponding esters [28,29,30] or amides [34,35,36,37,38,39].

Taking in consideration of previous works based on the Mannich bases of naphthol derivatives and the *ortho*-quinone methides (*o*Qm) derived from them [40], an extension of the synthetic procedures applying a possible special *o*Qm—derived from KYNA through its naphthol analogy and its Mannich bases [41]—was planned. As a base for these transformations, the syntheses of indole- and 2-naphthol-containing triarylmethane (TRAM) derivatives [42,43,44] were chosen. These TRAM derivatives could be synthesized through two similar pathways: (*i)* through the reaction of 2-naphthol and the Mannich base of indole or (*ii)* using the reaction of indole and the Mannich base of 2-naphthol. We hypothesized that similar reactions can be carried out in the case of KYNA as well, possibly yielding special bioconjugates.

## 2. Materials and Methods

^1^H and ^13^C-NMR spectra were recorded in DMSO-d_6_ and CDCl_3_ solutions in 5 mm tubes at room temperature (RT), on a Bruker DRX-500 spectrometer (Bruker Biospin, Karlsruhe, Baden Württemberg, Germany) at 500 (1H) and 125 (13C) MHz, with the deuterium signal of the solvent as the lock and TMS as internal standard (1H, 13C).

Melting points were determined on a Hinotek X-4 melting point apparatus. Merck Kieselgel 60F_254_ plates were used for TLC.

HRMS flow injection analysis was performed with Thermo Scientific Q Exactive Plus hybrid quadrupole-Orbitrap (Thermo Fisher Scientific, Waltham, MA, USA) mass spectrometer coupled to a Waters Acquity I-Class UPLC™ (Waters, Manchester, UK).

The synthesis of compounds **16a** was carried out according to literature method [45].

### 2.1. General Procedure for the Synthesis of ***7, 20a–h***

(A) 4-Oxo-1,4-dihydroquinoline-2-carboxylic acid ethyl ester (**6**) or its derivatives (**19a**–**h**, 0.1 mmol) and **4** (0.15 mmol) were placed in a pressure-resistant 10 mL vessel with toluene (5 mL). The mixture was kept at 160 °C for 3 h in a CEM Discover SP microwave reactor (300 W). Work-up is similar to method B) but lower conversions and yields have been achieved with method A).
(B) 4-Oxo-1,4-dihydroquinoline-2-carboxylic acid ethyl ester (**6**) or its derivatives (**19a**–**h**, 0.5 mmol) and **4** (0.75 mmol) were placed in a 50 mL round-bottom flask. The mixture was heated at reflux temperature in toluene (25 mL) for 1.5–18 h. Work-up is described separately.

#### 2.1.1. Ethyl 3-((1H-indol-3-yl)(phenyl)methyl)-4-oxo-1,4-dihydroquinoline-2-carboxylate (**7**)

Preparation according to general procedure, using **6**; reflux time: 1.5 h. Work-up: crystals formed after cooling the reaction, filtered, and washed with 10 mL toluene. Yield: 131 mg (62%); M.p. 275–277 °C. HRMS calcd for [M + H^+^] *m*/*z* = 423.1708, found *m*/*z* = 423.1702; ^1^H NMR (DMSO-d6); 0.99 (3H, t, J = 7.2 Hz); 3.79–3,98 (2H, m); 6.11 (1H, s); 6.85–6.90 (2H, m); 7.03 (2H, s); 7.12–7.18 (2H, m); 7.73 (1H, t, J = 8.1 Hz); 7.22 (2H, t, J = 7.6 Hz); 7.26–7.30 (2H, m); 7.31–7.38 (2H, m); 7.61 (1H, d, J = 8.3 Hz); 7.68 (1H, t, J = 7.6 Hz); 8.08 (1H, d, J = 8.2 Hz); 10.80 (1H, s); 11.94 (1H, s); ^13^C NMR (DMSO-d6); 13.8; 62.5; 111.8; 115.5; 118.6; 118.8; 121.2; 122.1; 124.0; 124.7; 125.6; 125.7; 126.0; 127.8; 128.0; 129.2; 132.7; 136.5; 139.4; 140.5; 143.2; 163.8; 176.4; (Figures S1 and S2); FTIR in Figure S3.

#### 2.1.2. Ethyl 3-((1H-indol-3-yl)(phenyl)methyl)-5-chloro-4-oxo-1,4-dihydroquinoline-2-carboxylate (**20a**)

Preparation according to general procedure, using **19a**; reflux time: 8 h. Work-up: after evaporation of solvent purified by column chromatography (eluent = n-hexane:EtOAc 1:2), crystallized from 10 mL Et_2_O, filtered, and washed with 10 mL Et_2_O. Yield: 208 mg (91%); M.p. 245–247 °C. HRMS calcd for [M + H^+^] *m*/*z* = 457.1318, found *m*/*z* = 457.1315; ^1^H NMR (DMSO-d6); 0.95 (3H, t, J = 7.2 Hz); 3.71–3,80 (1H, m); 3.81–3,91 (1H, m); 6.10 (1H, s); 6.83 (1H, s); 6.89 (1H, t, J = 7.5 Hz); 7.04 (1H, t, J = 7.5 Hz); 7.12–7.19 (2H, m); 7.20–7.31 (5H, m); 7.35 (1H, d, J = 8.1 Hz); 7.53–7.59 (2H, m); 10.81 (1H, s); 11.92 (1H, s); ^13^C NMR (DMSO-d6); 13.7; 62.5; 111.8; 115.3; 118.3; 118.6; 118.9; 120.6; 121.3; 123.8; 125.8; 126.1; 126.5; 127.8; 128.0; 129.2; 132.4; 132.6; 136.5; 139.7; 142.0; 143.0; 163.4; 175.6; (Figures S4 and S5); FTIR in Figure S6.

#### 2.1.3. Ethyl 3-((1H-indol-3-yl)(phenyl)methyl)-6-chloro-4-oxo-1,4-dihydroquinoline-2-carboxylate (**20b**)

Preparation according to general procedure, using **19b**; reflux time: 8 h. Work-up: after evaporation of solvent purified by column chromatography (eluent = n-hexane:EtOAc 1:2), crystallized from 10 mL Et_2_O, filtered, and washed with 10 mL Et_2_O. Yield: 189 mg (83%); M.p. decomposition over 260 °C. HRMS calcd for [M + H^+^] *m*/*z* = 457.1318, found *m*/*z* = 457.1316; ^1^H NMR (DMSO-d6); 0.99 (3H, t, J = 7.2 Hz); 3.81–3,90 (1H, m); 3.92–4.00 (1H, m); 6.09 (1H, s); 6.85–6.91 (2H, m); 7.04 (1H, t, J = 7.5 Hz); 7.11–7.19 (2H, m); 7.22 (2H, t, J = 7.5 Hz); 7.27 (2H, d, J = 8.4 Hz); 7.34 (1H, d, J = 8.4 Hz); 7.66 (1H, d, J = 8.9 Hz); 7.73 (1H, d, J = 9.1 Hz); 8.01 (1H, s); 10.82 (1H, s); 12.15 (1H, s); ^13^C NMR (DMSO-d6); 13,8; 62.7; 111.8; 115.2; 118.6; 118.8; 121.3; 121.4; 122.6; 124.6; 125.6; 125.7; 126.1; 127.7; 128.0; 128.7; 129.1; 132.9; 136.5; 138.0; 140.6; 143.0; 163.6; 175.4; (Figures S7 and S8) FTIR in Figure S9.

#### 2.1.4. Ethyl 3-((1H-indol-3-yl)(phenyl)methyl)-7-chloro-4-oxo-1,4-dihydroquinoline-2-carboxylate (**20c**)

Preparation according to general procedure, using **19c**; reflux time: 8 h. Work-up: after evaporation of solvent purified by column chromatography (eluent = n-hexane:EtOAc 1:2), crystallized from 10 mL Et_2_O, filtered, and washed with 10 mL Et_2_O. Yield: 174 mg (76%); M.p. 262–264 °C. HRMS calcd for [M + H^+^] *m*/*z* = 457.1318, found *m*/*z* = 457.1314; ^1^H NMR (DMSO-d6); 1.00 (3H, t, J = 7.2 Hz); 3.81–4.01 (2H, m); 6.11 (1H, s); 6.85–6.91 (2H, m); 7.04 (1H, t, J = 7.5 Hz); 7.12–7.18 (2H, m); 7.22 (1H, t, J = 7.5 Hz); 7.27 (1H, d, J = 7.4 Hz); 7.32–7.38 (2H, m); 7.66 (1H, s); 8.07 (1H, d, J = 8.8 Hz); 10.82 (1H, s); 12.01 (1H, s); ^13^C NMR (DMSO-d6); 13.8; 39.0; 62.7; 111.8; 115.2; 117.9; 118.6; 118.8; 121.3; 123.1; 123.3; 124.4; 125.7; 126.1; 127.7; 128.0; 128.1; 129.1; 129.2; 136.5; 137.4; 140.1; 140.6; 143.0; 163.6; 176.0; (Figures S10 and S11) FTIR in Figure S12.

#### 2.1.5. Ethyl 3-((1H-indol-3-yl)(phenyl)methyl)-8-chloro-4-oxo-1,4-dihydroquinoline-2-carboxylate (**20d**)

Preparation according to general procedure, using **19d**; reflux time: 8 h. Work-up: after evaporation of solvent purified by column chromatography (eluent = n-hexane:EtOAc 1:2), crystallized from 10 mL Et_2_O, filtered, and washed with 10 mL Et_2_O. Yield: 87 mg (38%); M.p. 219–221 °C. HRMS calcd for [M + H^+^] *m*/*z* = 457.1318, found *m*/*z* = 457.1309; ^1^H NMR (DMSO-d6); 0.98 (3H, t, J = 7.5 Hz); 3.85–4.02 (2H, m); 5.93 (1H, s); 6.88 (1H, t, J = 7.0 Hz); 6.92 (1H, s); 7.04 (1H, t, J = 7.5 Hz); 7.12–7.19 (2H, m); 7.22 (1H, t, J = 7.5 Hz); 7.29 (1H, d, J = 7.6 Hz); 7.32–7.38 (2H, m); 7.88 (1H, d, J = 7.6 Hz); 8.07 (1H, d, J = 8.8 Hz); 10.82 (1H, s); 11.34 (1H, s); ^13^C NMR (DMSO-d6); 13.7; 62.6; 111.8; 111.9; 114.9; 118.6; 118.8; 121.2; 121.9; 122.3; 124.3; 125.2; 125.7; 126.2; 126.3; 127.7; 128.0; 129.1; 132.9; 136.0; 136.5; 141.6; 142.9; 163.1; 176.0; (Figures S13 and S14) FTIR in Figure S15.

#### 2.1.6. Ethyl 3-((1H-indol-3-yl)(phenyl)methyl)-5-methoxy-4-oxo-1,4-dihydroquinoline-2-carboxylate (**20e**)

Preparation according to general procedure, using **19e**; reflux time: 8 h. Work-up: after evaporation of solvent purified by column chromatography (eluent = n-hexane:EtOAc 1:2), crystallized from 10 mL Et_2_O, filtered, and washed with 10 mL Et_2_O. Yield: 185 mg (81%); M.p. 160–163 °C. HRMS calcd for [M + H^+^] *m*/*z* = 453.1814, found *m*/*z* = 453.1804; ^1^H NMR (CDCl_3_); 1.04 (3H, t, J = 7.1 Hz); 3.91–4.01 (2H, m); 4.03 (1H, s); 6.28 (1H, s); 6.83 (1H, d, J = 7.9 Hz); 6.97 (1H, s); 7.01 (1H, t, J = 7.3 Hz); 7.16 (1H, t, J = 7.5 Hz); 7.20 (1H, d, J = 7.3 Hz); 7.23–7.25 (1H, m); 7.30–7.39 (1H, m); 7.53 (1H, t, J = 8.3 Hz); 7.70 (1H, d, J = 8.5 Hz); 8.01 (1H, s); 10.21 (1H, s); ^13^C NMR (DMSO-d6); 13.7; 38.8; 56.0; 62.3; 104.5; 110.3; 111.8; 115.3; 115.6; 118.6; 118.8; 121.2; 123.6; 125.7; 125.9; 127.8; 127.9; 129.1; 133.0; 136.5; 138.8; 142.2; 143.4; 160.0; 163.7; 175.9; (Figures S16 and S17) FTIR in Figure S18.

#### 2.1.7. Ethyl 3-((1H-indol-3-yl)(phenyl)methyl)-6-methoxy-4-oxo-1,4-dihydroquinoline-2-carboxylate (**20f**)

Preparation according to general procedure, using **19f**; reflux time: 5 h. Work-up: after evaporation of solvent purified by column chromatography (eluent = DCM:EtOH 100:2.5), crystallized from 10 mL Et_2_O, filtered, and washed with 10 mL Et_2_O. Yield: 133 mg (58%); M.p. decomposition over 180 °C. HRMS calcd for [M + H^+^] *m*/*z* = 453.1814, found *m*/*z* = 453.1805; ^1^H NMR (DMSO-d6); 1.01 (3H, t, J = 7.1 Hz); 3.82 (1H, s); 3.85–4.03 (2H, m); 6.13 (1H, s); 6.87 (1H, t, J = 7.4 Hz); 6.90 (1H, s); 7.03 (1H, t, J = 7.3 Hz); 7.15 (1H, d, J = 7.9 Hz); 7.21 (1H, t, J = 7.7 Hz); 7.29 (1H, d, J = 7.9 Hz); 7.31–7.36 (2H, m); 7.48 (1H, s); 7.59 (1H, d, J = 9.1 Hz); 10.79 (1H, s); 11.94 (1H, s); ^13^C NMR (DMSO-d6); 13.8; 55.8; 62.5; 104.6; 111.8; 115.6; 118.6; 118.8; 120.7; 121.2; 123.5; 125.6; 125.9; 125.9; 127.8; 127.9; 129.1; 134.0; 136.5; 139.4; 143.4; 156.3; 164.0; 175.7; (Figures S19 and S20) FTIR in Figure S21.

#### 2.1.8. Ethyl 3-((1H-indol-3-yl)(phenyl)methyl)-7-methoxy-4-oxo-1,4-dihydroquinoline-2-carboxylate (**20g**)

Preparation according to general procedure, using **19g**; reflux time: 18 h. Work-up: after evaporation of solvent purified by column chromatography (eluent = n-hexane:EtOAc 1:2), crystallized from 10 mL Et_2_O, filtered, and washed with 10 mL Et_2_O. Yield: 217 mg (95%); M.p. decomposition over 260 °C. HRMS calcd for [M + H^+^] *m*/*z* = 453.1814, found *m*/*z* = 453.1807; ^1^H NMR (DMSO-d6); 1.00 (3H, t, J = 7.0 Hz); 3.81–3.99 (5H, m); 6.12 (1H, s); 6.85–6.90 (2H, m); 6.94 (1H, d, J = 8.9 Hz); 7.00 (1H, s); 7.03 (1H, t, J = 7.7 Hz); 7.11–7.17 (2H, m); 7.21 (1H, t, J = 7.6 Hz); 7.27 (1H, d, J = 7.8 Hz); 7.34 (1H, d, J = 8.1 Hz); 7.97 (1H, d, J = 9.0 Hz); 10.79 (1H, s); 11.70 (1H, s); ^13^C NMR (DMSO-d6); 13.8; 38.9; 56.0; 62.4; 99.3; 111.8; 114.4; 115.6; 118.7; 118.8; 119.2; 121.2; 122.2; 125.6; 126.0; 127.6; 127.8; 127.9; 129.1; 136.5; 139.9; 141.2; 143.4; 162.7; 163.8; 175.9; (Figures S22 and S23) FTIR in Figure S24.

#### 2.1.9. Ethyl 3-((1H-indol-3-yl)(phenyl)methyl)-8-methoxy-4-oxo-1,4-dihydroquinoline-2-carboxylate (**20h**)

Preparation according to general procedure, using **19h**; reflux time: 8 h. Work-up: after evaporation of solvent purified by column chromatography (eluent = n-hexane:EtOAc 1:2), crystallized from 10 mL Et_2_O, filtered, and washed with 10 mL Et_2_O. Yield: 23 mg (10%); M.p. 244–246 °C. HRMS calcd for [M + H^+^] *m*/*z* = 453.1814, found *m*/*z* = 453.1804; ^1^H NMR (DMSO-d6); 0.95 (3H, t, J = 7.1 Hz); 3.79–3.96 (2H, m); 3.99 (3H, s); 5.95 (1H, s); 6.84–6.91 (2H, m); 7.02 (1H, t, J = 7.3 Hz); 7.11–7.17 (2H, m); 7.21 (2H, t, J = 7.8 Hz); 7.24–7.32 (4H, m); 7.34 (1H, d, J = 8.1 Hz); 7.61–7.66 (1H, m); 10.79 (1H, s); 11.45 (1H, s); ^13^C NMR (DMSO-d6); 13.7; 56.7; 62.3; 111.8; 111.8; 115.3; 116.9; 118.6; 118.7; 121.2; 121.5; 123.7; 125.6; 125.7; 126.1; 127.8; 128.0; 129.2; 130.2; 136.5; 140.9; 143.2; 149.0; 163.5; 176.0; (Figures S25 and S26) FTIR in Figure S27.

### 2.2. Procedure for the Synthesis of 2-(((2-(dimethylamino)ethyl)amino)(phenyl)methyl)naphthalen-1-ol (***16b***)

1-Naphthol (**14**, 1.0 mmol) *N,N*-dimethylethane-1,2-diamine (1.0 mmol) and benzaldehyde (1.0 mmol) were placed in a 50 mL round-bottom flask. The mixture was heated at reflux temperature in toluene (25 mL) for 30 min. After the evaporation of the solvent the residue was purified using silica column chromatography with an eluent of n-hexane:MeOH:DCM 3:1:1 mixture collecting the last compound. The solvent was evaporated leaving behind a brownish yellow viscous liquid. Yield: 192 mg (60%). HRMS calcd for [M + H^+^] *m*/*z* = 321.1967, found *m*/*z* = 321.1957; ^1^H NMR (CDCl_3_); 2.20 (6H, s); 2.36–2.43 (1H, m); 2.57–2.67 (1H, m); 2.79–2.87 (2H, m); 5.01 (1H, s); 6.97 (1H, d, J = 8.3 Hz); 7.19–7.27 (2H, m); 7.27–7.35 (3H, m); 7.35–7.41 (2H, m); 7.41–7,49 (2H, m); 7.71 (1H, d, J = 7.3 Hz); 8.30 (1H, d, J = 8.2 Hz); ^13^C NMR (CDCl_3_); 45.3; 45.5; 58.1; 68.4; 117.1; 118.3; 122.4; 124.8; 125.6; 126.1; 126.9; 127.3; 127.5; 127.8; 128.9; 142.2; 153.6; (Figures S28 and S29) FTIR in Figure S30.

### 2.3. Procedures for the synthesis of 2-((1H-indol-3-yl)(phenyl)methyl)naphthalen-1-ol (***18***)

1-Naphthol (**14**, 0.5 mmol) and **4** (0.75 mmol) were placed in a 50 mL round-bottom flask. The mixture was heated at reflux temperature in toluene (25 mL) for 8 h. After the evaporation of the solvent, the residue was purified by column chromatography (eluent = n-hexane:EtOAc, 4:1). Crystallization from 10 mL Et_2_O, washed with 10 mL Et_2_O. Yield: 122 mg (70%); M.p. decomposition over 160 °C [46]. HRMS calcd for [M - H^+^] *m*/*z* = 348.1388, found *m*/*z* = 348.1385; ^1^H NMR (DMSO-d6); 6.36 (1H, s); 6.59 (1H, s); 6.79 (1H, d, J = 7.8 Hz); 6.86–6.93 (2H, m); 7.09 (1H, t, J = 7.6 Hz); 7.17 (1H, d, J = 7.5 Hz); 7.21–7.27 (1H, m); 7.28–7.36 (4H, m); 7.41 (1H, d, J = 8.1 Hz); 7.45–7,49 (2H, m); 8.09–8.14 (1H, m); 8.20–8.25 (1H, m); 10.01 (1H, brs); 10.87 (1H, brs); ^13^C NMR (DMSO-d6); 23.3; 43.9; 67.8; 107.7; 112.0; 118.7; 118.8; 119.4; 121.5; 123.0; 124.4; 124.5; 125.1; 125.5; 126.4; 126.5; 127.0; 127.0; 128.3; 128.6; 129.2; 130.4; 132.8; 137.2; 144.9; 152.3; (Figures S31 and S32) FTIR in Figure S33.

## 3. Results and Discussion

**Scheme 1:**
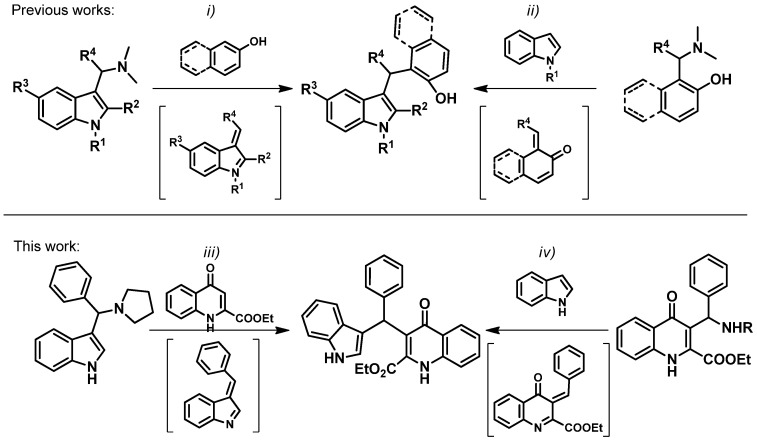
The synthesis of indole and 1-naphthol TRAMs (*i*) from indole-based Mannich products, (*ii*) naphthol-/phenol-based Mannich products and indole and KYNA TRAMs (*iii*) from the Mannich base of indole (*iv*) or the Mannich base of KYNA.

The work of Baruah et al. [42,43,44] includes both pathways of TRAM (**3**) synthesis starting either from indole-based Mannich products (Scheme 1, route i) or naphthol-/phenol-based Mannich products (route ii). Thus, the investigation of similar reaction routes is outlined for the ethyl ester of KYNA as well, starting from the Mannich base of indole (route iii) or the Mannich base of KYNA (route iv).

**Scheme 2:**
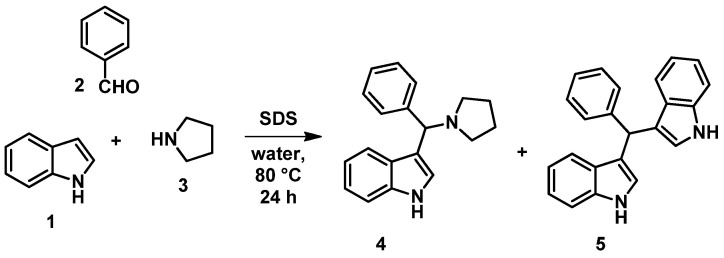
The synthesis of aminoalkylated indole derivatives.

First, based on route (iii), a reaction between the Mannich base of indole and KYNA ethyl ester was planned. To synthesize the Mannich base of indole (**4**), several literature methods have been explored. Reactions utilizing catalysts, such as ferric phosphate [47] and iodine [48], or protic solvents, such as ethanol, methanol [49], and ethylene glycol [50], resulted mainly in bisindole derivative **5** mentioned in the corresponding literature as a byproduct. However, Mannich base **4** could be synthesized under neat conditions applying only indole, benzaldehyde, and pyrrolidine (with or without L-proline as catalyst [51,52]). Surprisingly, the highest yield was achieved by using the application of the surfactant sodium dodecyl sulfate (SDS, Scheme 2) [53].

**Scheme 3:**
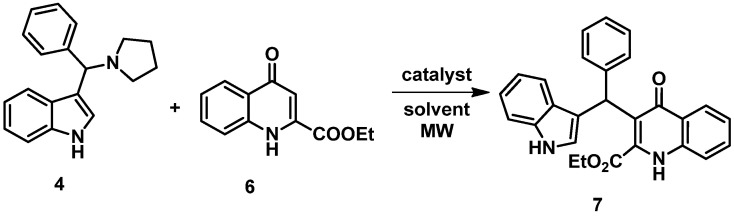
Synthesis of KYNA TRAMs through indole-based Mannich products.

With the starting material in our hands, in the first C–C bond-forming reactions, **4** was reacted with the ethyl ester of KYNA (**6**) in MeCN at 100 °C (under MW conditions) with thiourea as the catalyst, based on the work of Baruah et al. [42] (Scheme 3). The conversion determined by NMR spectrometry was low; thus, the best conditions used for the alternative reaction route [43] were investigated, showing promising results (Table 1). Fortunately, the still low yield of **7** could be further improved by raising the reaction temperature to 160 °C. However, any further increase caused a decomposition of the starting materials.

**Scheme 4:**
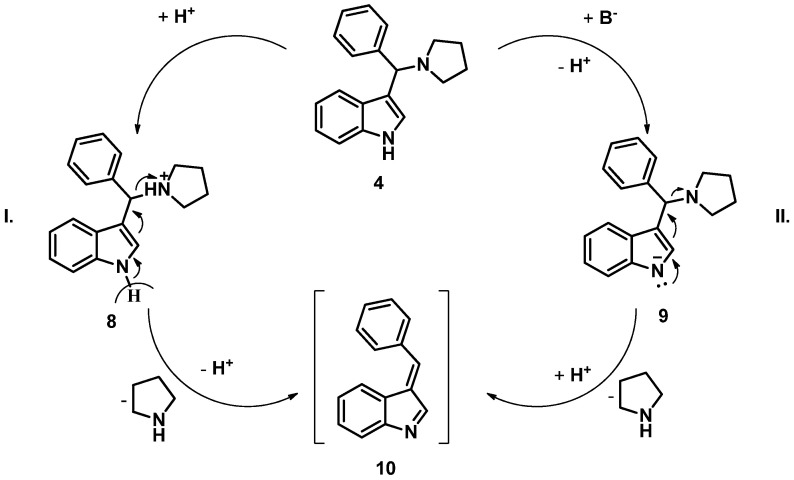
Proposed mechanisms in the case of acid (**I**) or base (**II**) catalysis.

The use of a catalyst was crucial, as the desired TRAM did not form without the use of a base or an acid catalyst. It is interesting to mention that although acid catalysis resulted in somewhat higher conversion, both acid and base catalysis could enhance the synthesis of **7**. Baruah et al. hypothesized that the reaction taking place between the indole derivative and varied electron-rich aromatic structures involves the formation of intermediate **10** [42,43]. In their proposed elimination–addition pathway starting from a Mannich base of indole, thiourea activates the amine moiety of the aminoalkyl function through double hydrogen bonding and converts it into a better leaving group (Scheme 4, I). Concerning triethylamine (TEA) used in our reactions, a hydrogen bond is unable to form; therefore, a more direct form of catalysis is proposed. Through the application of high temperature and TEA, the deprotonation of the indole moiety takes place followed by a subsequent rearrangement of the indole anion into benzylidene intermediate **10**. Then, the latter is attacked by a molecule of the electron-rich KYNA yielding compound **7**. It is also surmised that the C–N bond cleavage of the indole derivative could also take place through the elimination of pyrrolidine via the protonation of the amine moiety, making it a better leaving group and leading to intermediate **10** (Scheme 4, II).

Further optimization of the reaction involved the change of solvent from the aprotic and apolar toluene to solvents representing a wider range of the aprotic–protic and apolar–polar scale (Table 1). It is hypothesized that toluene may be the best solvent because of the lack of H-bridge bonds and polarity of the solvent can contribute to a more unstable, and thus more reactive, intermediate [54].

**Scheme 5:**
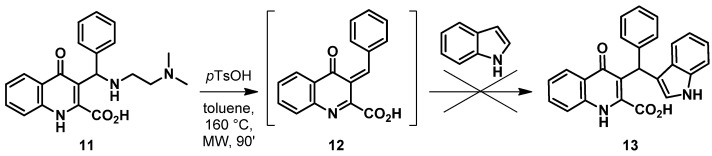
Synthesis of KYNA TRAM through aminoalkylated KYNA.

After successfully optimizing the reaction through route (iii), the synthesis of **7** was planned through the reaction of the KYNA Mannich base with indole (Scheme 1, route iv). KYNA Mannich derivatives synthesized previously are abundant [41]; however, compounds containing the crucial phenol structure were narrowed down only to a single compound (**11**, Scheme 5). Unfortunately, using this derivative in the reaction under conditions optimized previously did not result in the desired compound.

**Scheme 6:**
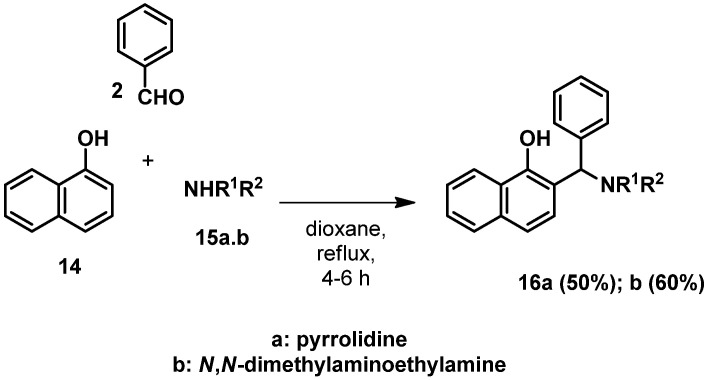
Synthesis of aminoalkylated 1-naphthol derivatives **16a**,**b**.

It is presumed that this may be due to the *N,N*-dimethylaminoethyl moiety being a bad leaving group. In order to fully support this hypothesis, a synthetic procedure was applied. Unfortunately, a Mannich base of KYNA containing a secondary amine function could not be synthesized, which is probably due to steric hindrance. Thus, considering the similarity of 1-naphthol to KYNA [41], the synthesis of Mannich bases **16a** and **16b** was carried out, as shown in Scheme 6.

**Scheme 7:**
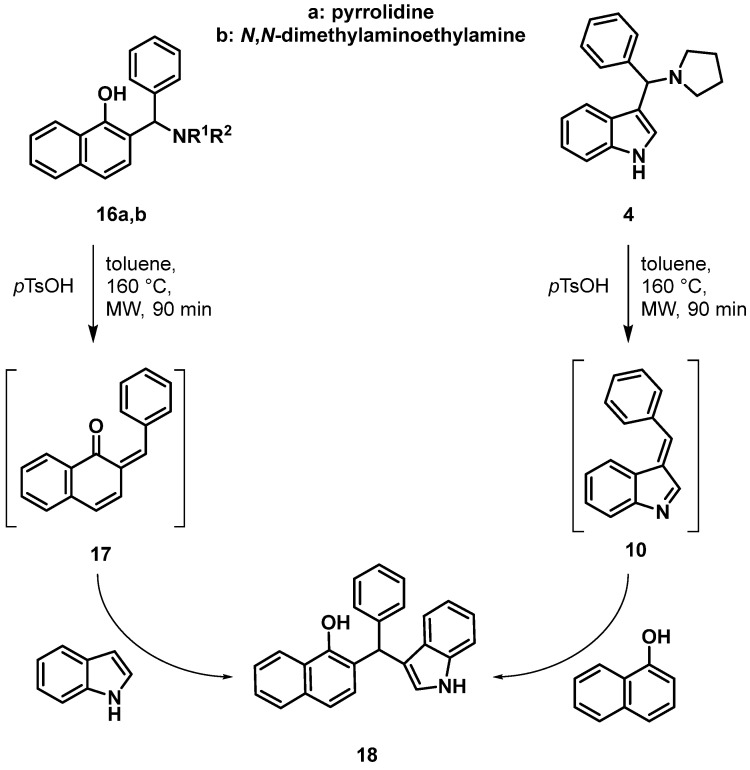
Comparison of the syntheses of **18**. Conversions: starting from **16a** 10%, **16b** ~1 %, and **4** 99%. Yield of **18** starting from **4**: 70%.

A comparison of the reaction of 1-naphthol with **6** and the reactions of **16a** and **16b** with indole (Scheme 7) allows us to arrive at two conclusions: (a) Mannich bases containing secondary amines (e.g., **11** and **16b**) are less prone to undergo the transformation because of a bad leaving group character; (b) reactions through intermediate **10** are more preferable compared to reactions via possible ortho-quinone methide intermediates **12** and **17** derived either from **11** or from the Mannich bases of 1-naphthol (**16a**,**b**). This may be due to a possible hydrogen bridge between the hydroxy/oxo group in **16a**,**b** and **11** in addition to the amine moiety, making the protonated form a more stable intermediate.

**Scheme 8:**
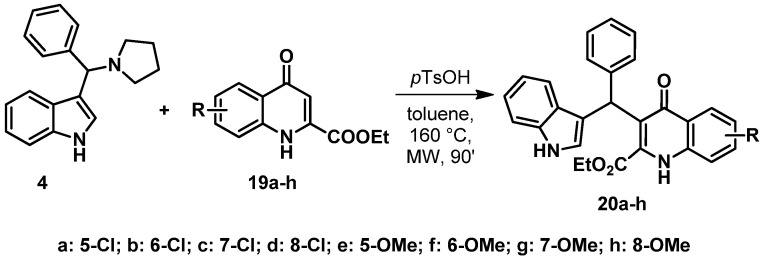
Synthesis of TRAMs **20a**–**h** via aminoalkylated indole.

To further investigate the scope and limitations of the reaction, the synthesis of TRAMs containing different KYNA derivatives was planned. The reactions were carried out applying the optimized conditions (see Table 1, Entry #4) starting from KYNA derivatives **19a**–**h** substituted at the B ring (Scheme 8). The reactions resulted in a diverse range of compounds (**20a**–**h**).

In the case of derivative **19e**, the reaction applying microwaves as a heat source resulted in an exceptionally low conversion. To test whether a kinetic control takes place during the transformation, a longer reflux treatment was carried out. As the result with an almost full conversion was promising, reflux conditions were applied to the other derivatives as well, showing a general increase in conversions and supporting our hypothesis.

It is interesting to mention that the type of substituents on the B ring influenced the reactions to a lesser extent (e.g., Table 2, Entry #2, and #10) compared to the position of the substituents (e.g., Table 2, #10, and #16). Both chloro- and methoxy-KYNA derivatives, with substituents at C–5 and C–7, showed somewhat lower reactivity compared to the ethyl ester of KYNA (longer reaction times were needed). However, the same substituents in positions C–6 and C–8 caused a significant decrease in reactivity of the KYNA skeleton.

## 4. Conclusions

The synthesis of TRAM bioconjugates consisting of indole and the ethyl ester of kynurenic acid has been accomplished. The reactions take place through the cleavage of the C–N bond of indole Mannich base and subsequent C–C bond formation between the benzylidene intermediate and KYNA. To further investigate the scope and limitations of the reaction, KYNA derivatives bearing chloro and methoxy groups were also reacted yielding a wide variety of new TRAM KYNA derivatives with possible bioactivities. On the basis of acid and base catalysis, two possible catalytic pathways are hypothesized, both promoting the elimination of the amine moiety. To investigate the reactivity of ortho-quinone methides (oQm), an alternative reaction route via the Mannich bases of KYNA and its structural analogue 1-naphthol was also investigated. This showed surprisingly no conversion through the possible—and highly reactive—oQm intermediate. This could be contributed to the lack of elimination of the amine moiety, due to it having a bad leaving group character. Thus, upon comparison with the reactions applying the indole Mannich base, a prominent tilt toward the synthesis involving the benzylidene intermediate was observed.

## Figures and Tables

**Table 1:** Optimization of the synthesis of **7**.

▪ Entry #	Solvent	Catalyst	Temperature (°C)	Time (min.)	Conversion (%) ^a^
▪ 1	MeCN	thiourea	100	10	5
▪ 2	toluene	*p*TsOH	100	180	20
▪ 3	toluene	*p*TsOH	130	180	60
▪ 4	toluene	*p*TsOH	160	90	80 ^b^
▪ 5	toluene	*-*	160	90	5
▪ 6	toluene	thiourea	160	90	40
▪ 7	toluene	L-proline	160	90	65
▪ 8	toluene	TEA	160	90	70
▪ 9	1,2-dichlorobenzene	*p*TsOH	160	90	0
▪ 10	MeCN	*p*TsOH	160	90	10
▪ 11	anisole	*p*TsOH	160	90	5
▪ 12	EtOH	*p*TsOH	160	90	0
▪ 13	water	SDS	160	60	0

^a^ = determined from crude NMR spectra. ^b^ = work-up performed to isolate **12** (yield: 62%).

**Table 2:** Comparison of the reactivity of substituted KYNA derivatives **19a**–**h**.

▪ Entry #	Starting Material	Temperature	Time (h)	Conversion (%) ^a^	Yield (%)
▪ 1	**19a**	160	3	60	44
▪ 2		reflux	8	99	91
▪ 3	**19b**	160	3	60	49
▪ 4		reflux	8	90	83
▪ 5	**19c**	160	3	70	62
▪ 6		reflux	8	85	76
▪ 7	**19d**	160	3	60	53
▪ 8		reflux	8	50	38
▪ 9	**19e**	160	3	30	18
▪ 10		reflux	8	90	81
▪ 11	**19f**	160	3	45	34
▪ 12		reflux	8	70	58
▪ 13	**19g**	160	3	60	48
▪ 14		reflux	5	99	95
▪ 15	**19h**	160	3	10	5
▪ 16		reflux	18	20	10

^a^ = determined from crude NMR spectra.

## Data Availability

Not applicable.

## Supplementary Materials

The ^1^H, ^13^C NMR spectra, FTIR spectra for all compounds and 2D NMR spectra for compound **7** as supporting information can be downloaded at: https://www.mdpi.com/article/10.3390/ijms23137152/s1.

## References

[1] Sundberg R.J. (1996). Indoles.

[2] Gribble G.W. (2010). Heterocyclic Chemistry II.

[3] Yang T., Lu H., Shu Y., Ou Y., Hong L., Au C.T., Qiu R. (2020). CF_3_SO_2_Na-Mediated, UV-Light-Induced Friedel–Crafts Alkylation of Indoles with Ketones/Aldehydes and Bioactivities of Products. Org. Lett..

[4] Humphrey G.R., Kuethe J.T. (2006). Practical methodologies for the synthesis of indoles. Chem. Rev..

[5] Cacchi S., Fabrizi G. (2005). Synthesis and Functionalization of Indoles Through Palladium-catalyzed Reactions. Chem. Rev..

[6] Bartoli G., Bencivenni G., Dalpozzo R. (2010). Organocatalytic strategies for the asymmetric functionalization of indoles. Chem. Soc. Rev..

[7] Bandini M., Eichholzer A. (2009). Catalytic Functionalization of Indoles in a New Dimension. Angew. Chem. Int. Ed..

[8] Shiri M. (2012). Indoles in Multicomponent Processes (MCPs). Chem. Rev..

[9] Szatmári I., Sas J., Fülöp F. (2013). Catalyst-free coupling of indole derivatives with 3,4-dihydroisoquinoline and related compounds. Tetrahedron Lett..

[10] Takeda S., Yamamoto A., Okada T., Matsumura E., Nose E., Kogure K., Kojima S., Haga T. (2003). Identification of surrogate ligands for orphan G protein-coupled receptors. Life Sci..

[11] Nikaido Y., Koyama Y., Yoshikawa Y., Furuya T., Takeda S. (2015). Mutation analysis and molecular modeling for the investigation of ligand-binding modes of GPR84. J. Biochem..

[12] Fares F. (2014). The anti-carcinogenic effect of indole-3-carbinol and 3, 3′-diindolylmethane and their mechanism of action. Med. Chem..

[13] Kiselev V.I., Drukh V.M., Muyzhnek E.L., Kuznetsov I.N., Pchelintseva O.I., Paltsev M.A. (2014). Preclinical antitumor activity of the diindolylmethane formulation in xenograft mouse model of prostate cancer. Exp. Oncol..

[14] Pillaiyar T., Köse M., Sylvester K., Weighardt H., Thimm D., Borges G., Förster I., Kügelgen I., Müller C.E. (2017). Diindolylmethane Derivatives: Potent Agonists of the Immunostimulatory Orphan G Protein-Coupled Receptor GPR84. J. Med. Chem..

[15] Safe S., Papineni S., Chintharlapalli S. (2008). Cancer chemotherapy with indole-3-carbinol, bis(3′-indolyl)methane and synthetic analogs. Cancer Lett..

[16] Kramer H.-J., Podobinska M., Bartsch A., Battmann A., Thoma W., Bernd A., Kummer W., Irlinger B., Steglich W., Mayser P. (2005). Malassezin, a novel agonist of the aryl hydrocarbon receptor from the yeast Malassezia furfur, induces apoptosis in primary human melanocytes. ChemBioChem.

[17] Queiroz M.M.F., Queiroz E.F., Zeraik M.L., Ebrahimi S.N., Marcourt L., Cuendet M., Castro-Gamboa I., Hamburger M., Bolzani V.S., Wolfender J.-L. (2014). Chemical composition of the bark of Tetrapterys mucronata and identification of acetylcholinesterase inhibitory constituents. J. Nat. Prod..

[18] Liu X., Ma S., Toy P.H. (2019). Halogen Bond-Catalyzed Friedel-Crafts Reactions of Aldehydes and Ketones Using a Bidentate Halogen Bond Donor Catalyst: Synthesis of Symmetrical Bis-(indolyl)methanes. Org. Lett..

[19] Pillaiyar T., Gorska E., Schnakenburg G., Müller C.E. (2018). General Synthesis of Unsymmetrical 3,3′-(Aza)diindolylmethane Derivatives. J. Org. Chem..

[20] Xiao J., Wen H., Wang L., Xu L.B., Hao Z.H., Shao C.-L., Wang C.-Y. (2016). Catalyst-free dehydrative SN1-type reaction of indolyl alcohols with diverse nucleophiles on water. Green Chem..

[21] Huo C.D., Sun C.G., Wang C., Jia X.D., Chang W.J. (2013). Triphenylphosphine-m-sulfonate/Carbon Tetrabromide as an Efficient and Easily Recoverable Catalyst System for Friedel–Crafts Alkylation of Indoles with Carbonyl Compounds or Acetals. ACS Sustain. Chem. Eng..

[22] Bayindir S., Saracoglu N. (2016). A facile one-pot method to synthesise 2-alkylated indole and 2,2′-bis(indolyl)methane derivatives using ketones as electrophiles and their anion sensing ability. RSC Adv..

[23] Sas K., Robotka H., Toldi J., Vécsei L. (2007). Mitochondria, metabolic disturbances, oxidative stress and the kynurenine system, with focus on neurodegenerative disorders. J. Neurol. Sci..

[24] Gigler G., Szénási G., Simó A., Lévay G., Hársing L.G., Sas K., Vécsei L., Toldi J. (2007). Neuroprotective effect of L-kynurenine sulfate administered before focal cerebral ischemia in mice and global cerebral ischemia in gerbils. Eur. J. Pharmacol..

[25] Luchowska E., Luchowski P., Sarnowska A., Wielosz M., Turski W.A., Urbańska E.M. (2003). Endogenous level of kynurenic acid and activities of kynurenine aminotransferases following transient global ischemia in the gerbil hippocampus. Pol. J. Pharmacol..

[26] Harrison B.L., Baron B.M., Cousino D.M., McDonald I.A. (1990). 4-[(Carboxymethyl)oxy]- and 4-[(carboxymethyl)amino]-5,7-dichloroquinoline-2-carboxylic acid: New antagonists of the strychnine-insensitive glycine binding site on the N-methyl-D-aspartate receptor complex. J. Med. Chem..

[27] Edmont D., Rocher R., Plisson C., Chenault J. (2000). Synthesis and evaluation of quinoline carboxyguanidines as antidiabetic agents. Bioorg. Med. Chem. Lett..

[28] Bonina F.P., Arenare L., Ippolito R., Boatto G., Battaglia G., Bruno V., de Caprariis P. (2000). Synthesis, pharmacokinetics and anticonvulsant activity of 7-chlorokynurenic acid prodrugs. Int. J. Pharm..

[29] Manfredini S., Pavan B., Vertuani S., Scaglianti M., Compagnone D., Biondi C., Scatturin A., Tanganelli S., Ferraro L., Prasad P. (2002). Design, synthesis and activity of ascorbic acid prodrugs of nipecotic, kynurenic and diclophenamic acids, liable to increase neurotropic activity. J. Med. Chem..

[30] Manfredini S., Vertuani S., Pavan B., Vitali F., Scaglianti M., Bortolotti F., Biondi C., Scatturin A., Prasad P., Dalpiaz A. (2004). Design, synthesis and in vitro evaluation on HRPE cells of ascorbic and 6-bromoascorbic acid conjugates with neuroactive molecules. Bioorgan. Med. Chem..

[31] Stone T.W. (2000). Development and therapeutic potential of kynurenic acid and kynurenine derivatives for neuroprotection. Trends Pharmacol. Sci..

[32] Stone T.W. (2000). Inhibitors of the kynurenine pathway. Eur. J. Med. Chem..

[33] Nichols A.C., Yielding K.L. (1998). Anticonvulsant activity of 4-urea-5,7-dichlorokynurenic acid derivatives that are antagonists at the NMDA-associated glycine binding site. Mol. Chem. Neuropathol..

[34] Füvesi J., Somlai C., Németh H., Varga H., Kis Z., Farkas T., Károly N., Dobszay M., Penke Z., Penke B. (2004). Comparative study on the effects of kynurenic acid and glucosamine-kynurenic acid. Pharmacol. Biochem. Behav..

[35] Zhang L., Sun F., Li Y., Sun X., Liu X., Huang Y., Zhang L.H., Ye X.S., Xiao J. (2007). Rapid synthesis of iminosugar derivatives for cell-based in situ screening: Discovery of “hit” compounds with anticancer activity. ChemMedChem..

[36] Brik A., Lin Y.C., Elder J., Wong C.H. (2002). A quick diversity-oriented amide-forming reaction to optimise P-subsite residues of HIV protease inhibitors. Chem. Biol..

[37] Tossi A., Benedetti F., Norbedo S., Skrbec D., Berti F., Romeo D. (2003). Small hydroxyethylene-based peptidomimetics inhibiting both HIV-1 and C. albicans aspartic proteases. Bioorg. Med. Chem..

[38] Knyihár-Csillik E., Mihály A., Krisztin-Péva B., Robotka H., Szatmári I., Fülöp F., Toldi J., Csillik B., Vécsei L. (2008). The kynurenate analog SZR-72 prevents the nitroglycerol-induced increase of c-Fos immunoreactivity in the rat caudal trigeminal nucleus: Comparative studies of the effects of SZR-72 and kynurenic acid. Neurosci. Res..

[39] Fülöp F., Szatmári I., Vámos E., Zádori D., Toldi J., Vécsei L. (2009). Syntheses, transformations and pharmaceutical applications of kynurenic acid derivatives. Curr. Med. Chem..

[40] Barta P., Fülöp F., Szatmári I. (2018). Mannich base-connected syntheses mediated by *ortho*-quinone methides. Beilstein J. Org. Chem..

[41] Lőrinczi B., Csámpai A., Fülöp F., Szatmári I. (2020). Synthesis of New C-3 Substituted Kynurenic Acid Derivatives. Molecules.

[42] Deb M.L., Das C., Deka B., Saikla P.J., Baruah P.K. (2016). Hydrogen-Bond-Catalyzed Arylation of 3-(Aminoalkyl)indoles via C–N Bond Cleavage with Thiourea under Microwave Irradiation: An Approach to 3-(α,α-Diarylmethyl)indoles. Synlett.

[43] Deb M.L., Pegu C.D., Deka B., Dutta P., Kotmale A.S., Baruah P.K. (2016). Brønsted-Acid-Mediated Divergent Reactions of Betti Bases with Indoles: An Approach to Chromeno[2,3-b]indoles through Intramolecular Dehydrogenative C2-Alkoxylation of Indole. Eur. J. Org. Chem..

[44] Pegu C.D., Nasrin S.B., Deb M.L., Das D.J., Saikia K.K., Baruah P.K. (2017). CAN-Catalyzed microwave promoted reaction of indole with betti bases under solvent-free condition and evaluation of antibacterial activity of the products. Synth. Commun..

[45] Saidi M.R., Azizi N., Naimi-Jamal M.R. (2001). Lithium perchlorate assisted one-pot three-component aminoalkylation of electron-rich aromatic compounds. Tetrahedron Lett..

[46] Zhu P., Yi Y., Liu Y., Peng Y. (2022). Asymmetric Addition of α-Diazomethylphosphonate to Alkylideneindolenine Catalyzed by a Trifunctional BINAP-Based Monophosphonium Salt. Org. Lett..

[47] Aghaalikhani S., Behbahani F.K. (2016). Three-Component Synthesis of 3-Aminoalkylindoles using Iron(III) Phosphate. ChemistrySelect.

[48] Depa N., Erothu H. (2019). One-Pot Three-Component Synthesis of 3-Aminoalkyl Indoles Catalyzed by Molecular Iodine. ChemistrySelect.

[49] Cao J.-F., Chen Y.-L., Guan Z., He Y.-H. (2014). Catalyst-free, Solvent-promoted and Scalable Multicomponent Synthesis of 3-Aminoalkylated Indoles via a Mannich-type Reaction. Z. Nat. B.

[50] Rajesh C., Kholiya R., Pavan V.S., Rawat D.S. (2014). Catalyst-free, ethylene glycol promoted one-pot three component synthesis of 3-amino alkylated indoles via Mannich-type reaction. Tetrahedron Lett..

[51] Kumar A., Gupta M.K., Kumar M. (2012). L-Proline catalysed multicomponent synthesis of 3-amino alkylated indoles via a Mannich-type reaction under solvent-free conditions. Green Chem..

[52] Deb M.L., Baruah P.K. (2016). Deamination of Indole Mannich Bases: An Efficient Route to 3-Benzyl/Alkylindoles via a Metal-Free Transfer Hydrogenation Under Microwave Irradiation. Curr. Organocatal..

[53] Kumar A., Gupta M.K., Kumar M., Saxena D. (2013). Micelle promoted multicomponent synthesis of 3-amino alkylated indoles via a Mannich-type reaction in water. RSC Adv..

[54] Lőrinczi B., Csámpai A., Fülöp F., Szatmári I. (2021). Synthetic- and DFT modelling studies on regioselective modified Mannich reactions of hydroxy-KYNA derivatives. RSC Adv..

