# Supplementary material for: Synthesis of Indole-Coupled KYNA Derivatives via C–N Bond Cleavage of Mannich Bases
Source: Int J Mol Sci. 2022 Jun 28;23(13):7152. doi: 10.3390/ijms23137152 (PMC9266300; doi:10.3390/ijms23137152)
Supplement: Supplementary file 1 [file ijms-23-07152-s001.zip › ijms-1768040-supplementary.pdf]

Supplementary Information

Synthesis of indole-coupled KYNA derivatives *via* C–N bond cleavage of Mannich bases

Bálint Lőrinczi[1,2]; Péter Simon[1,2] and István Szatmári[1,2,3*]

[1] Institute of Pharmaceutical Chemistry, University of Szeged, Eötvös u. 6, H-6720 Szeged, Hungary; lorinczi.balint@szte.hu

[2] Institute of Pharmaceutical Chemistry, Interdisciplinary Excellence Center, University of Szeged, H-6720 Szeged, Hungary

[3] MTA-SZTE Stereochemistry Research Group, Hungarian Academy of Sciences, Eötvös u. 6, H-6720 Szeged, Hungary

* Correspondence: szatmari.istvan@szte.hu

Contents

1. ^{1}H NMR, ^{13}C NMR and FTIR spectra of synthesized compounds .... 2.
2. 2D NMR spectra of compound **7** .... 24.

Ethyl 3-((1H-indol-3-yl)(phenyl)methyl)-4-oxo-1,4-dihydroquinoline-2-carboxylate (**7**)

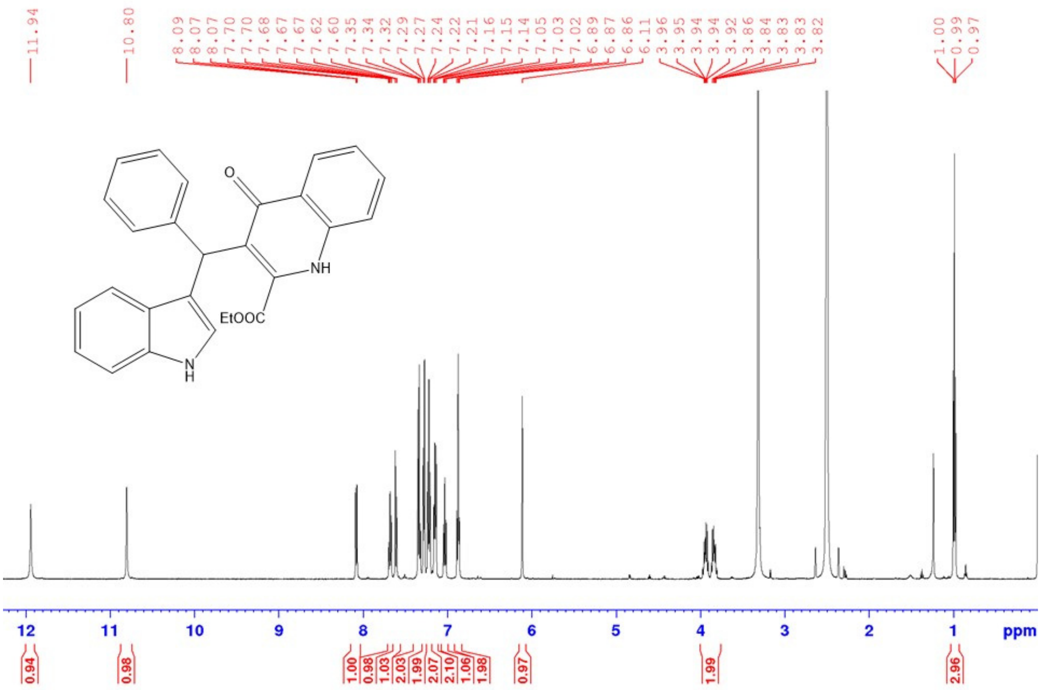

Figure S1. ^{1}H-NMR spectrum of **7**

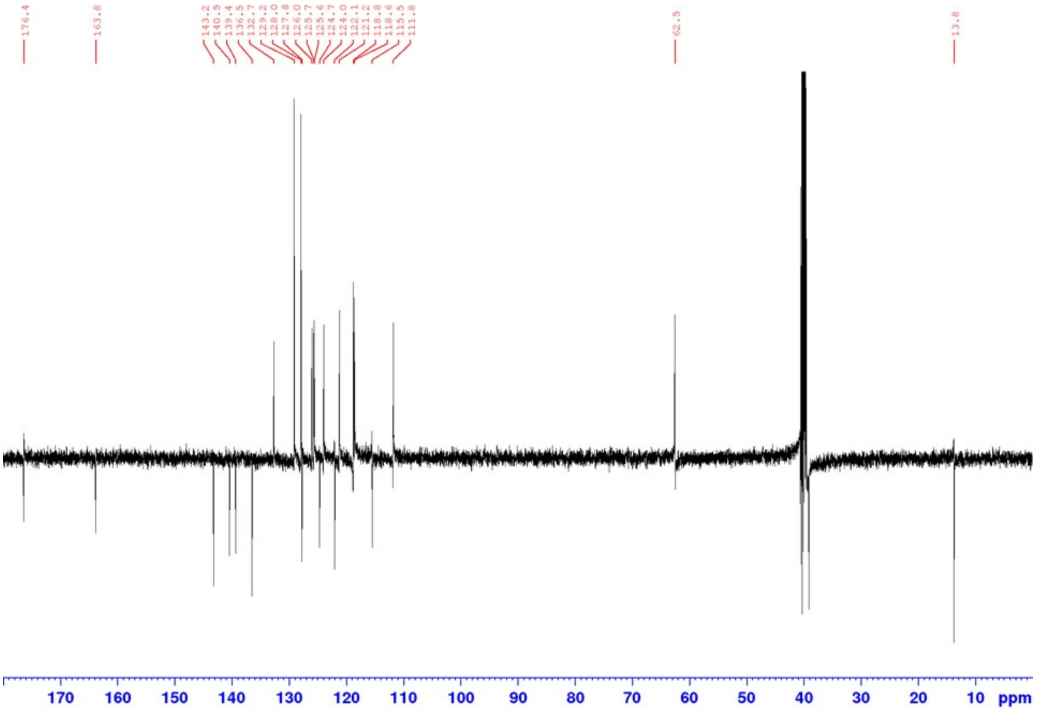

Figure S2. ^{13}C-NMR spectrum of **7**

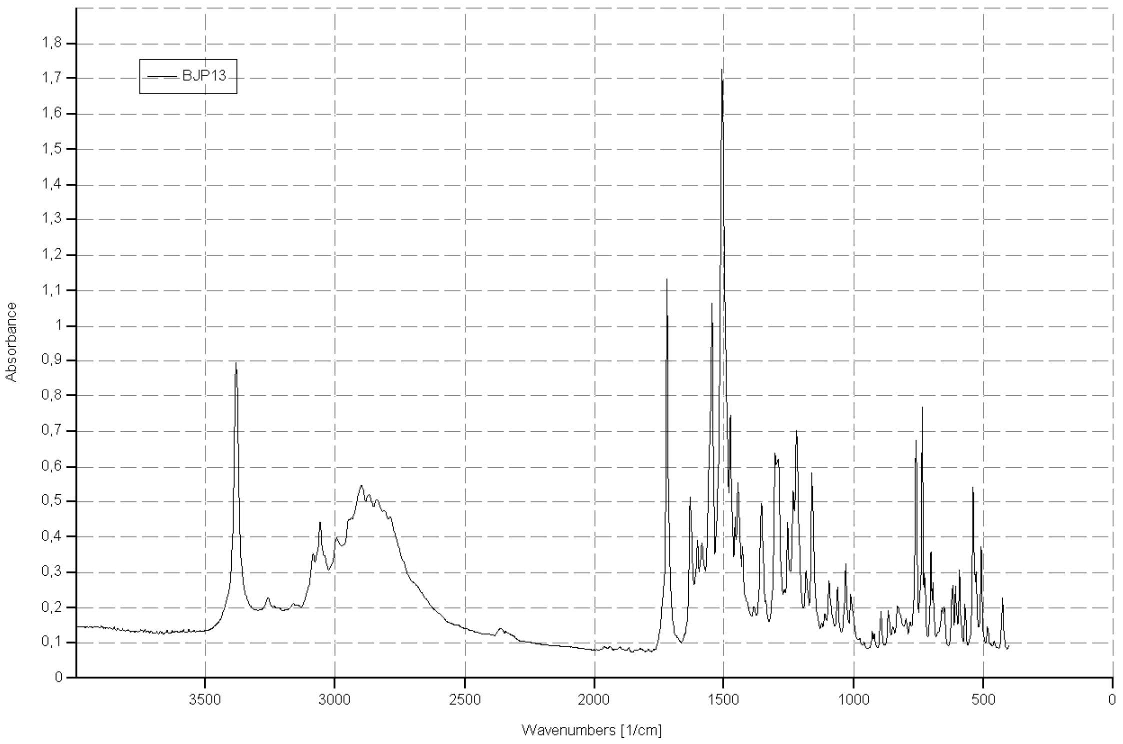

Figure S3. FTIR spectrum of **7**

Ethyl 3-((1H-indol-3-yl)(phenyl)methyl)-5-chloro-4-oxo-1,4-dihydroquinoline-2-carboxylate (**20a**)

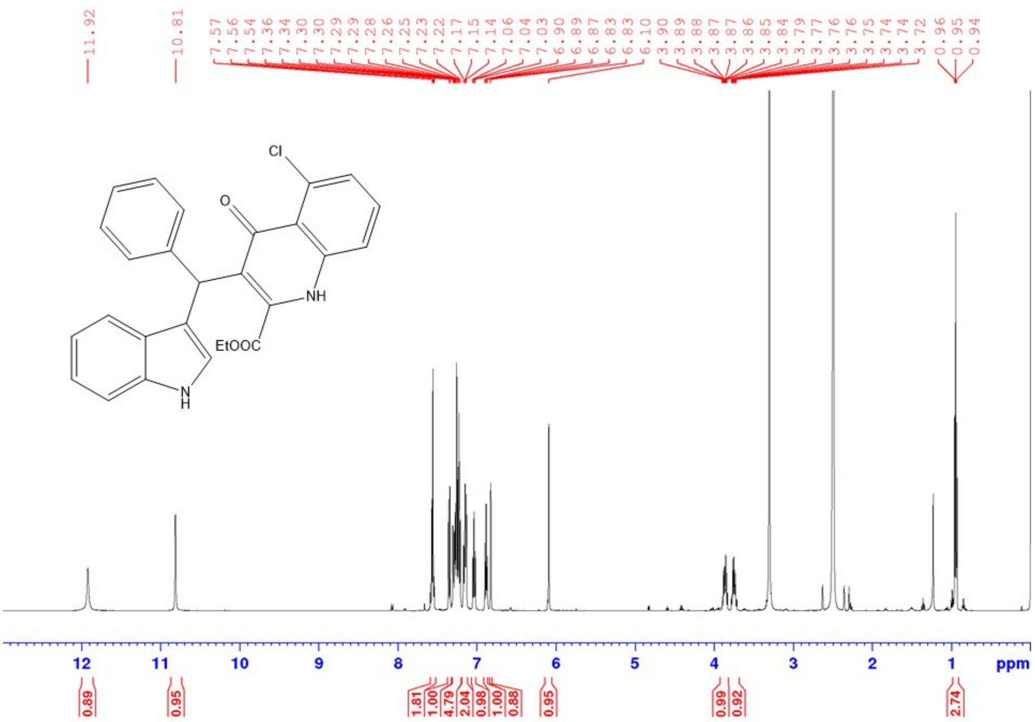

Figure S4. ^{1}H-NMR spectrum of **20a**

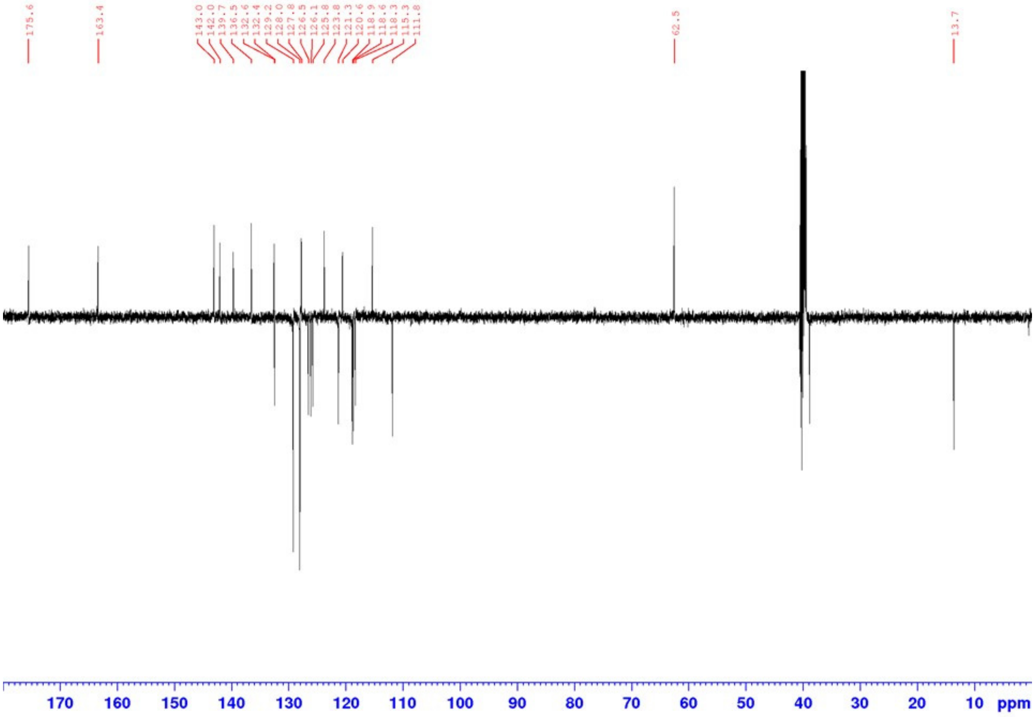

Figure S5. ^{13}C-NMR spectrum of **20a**

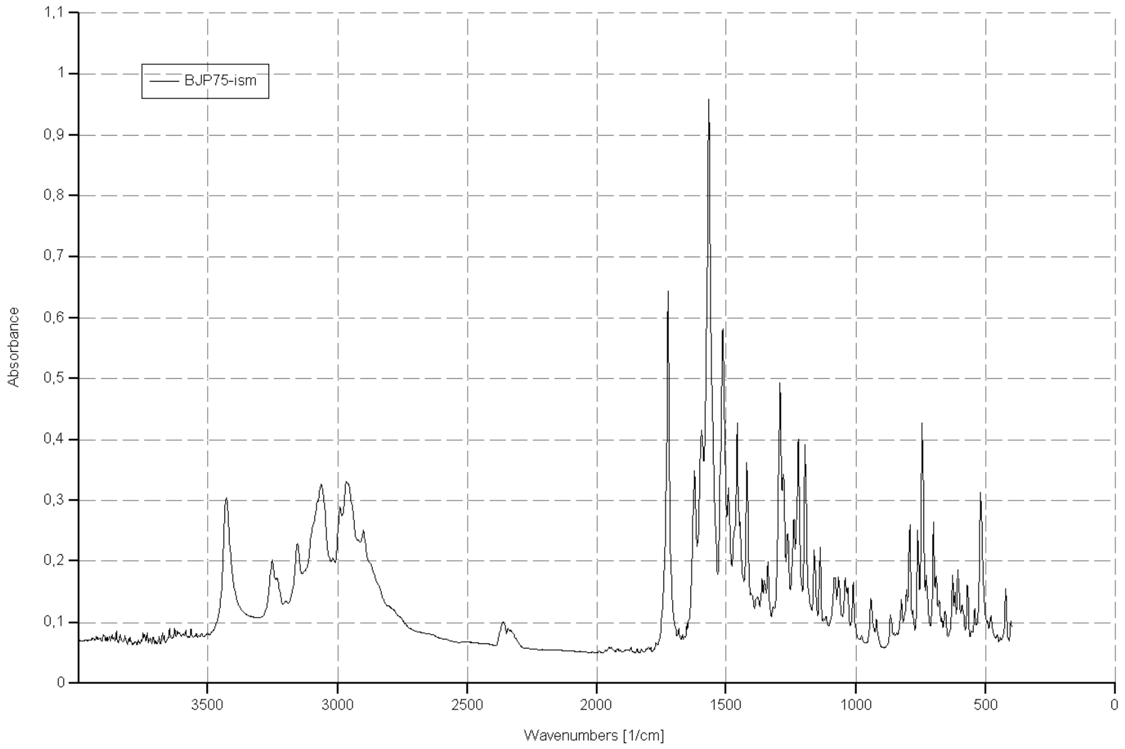

Figure S6. FTIR spectrum of **20a**

Ethyl 3-((1H-indol-3-yl)(phenyl)methyl)-6-chloro-4-oxo-1,4-dihydroquinoline-2-carboxylate (**20b**)

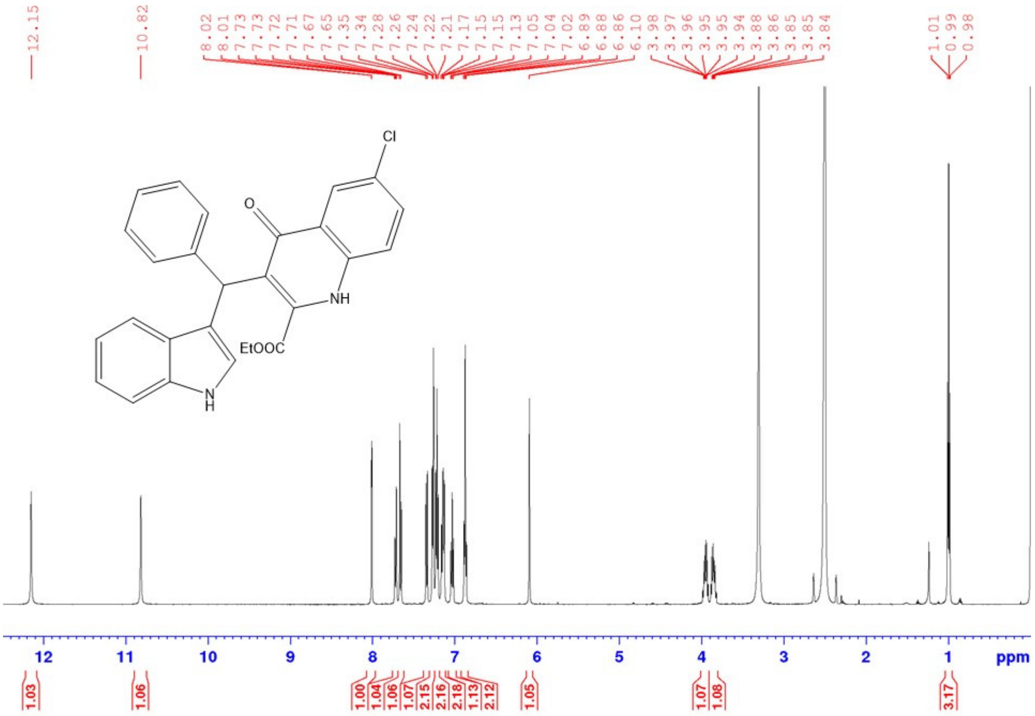

Figure S7. ^{1}H-NMR spectrum of **20b**

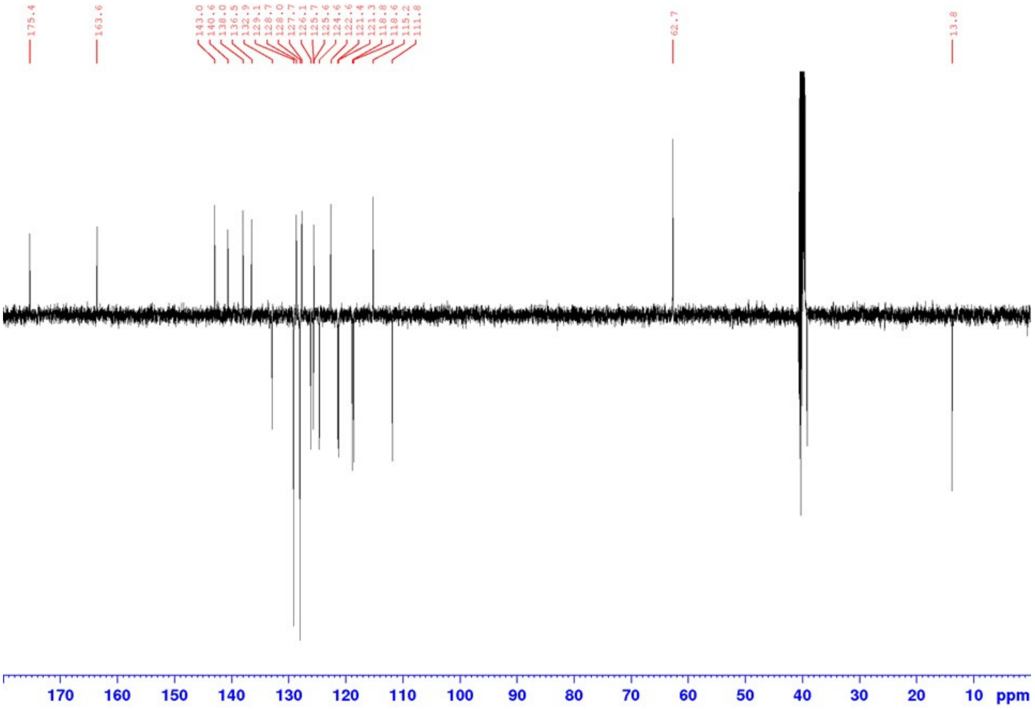

Figure S8. ^{13}C-NMR spectrum of **20b**

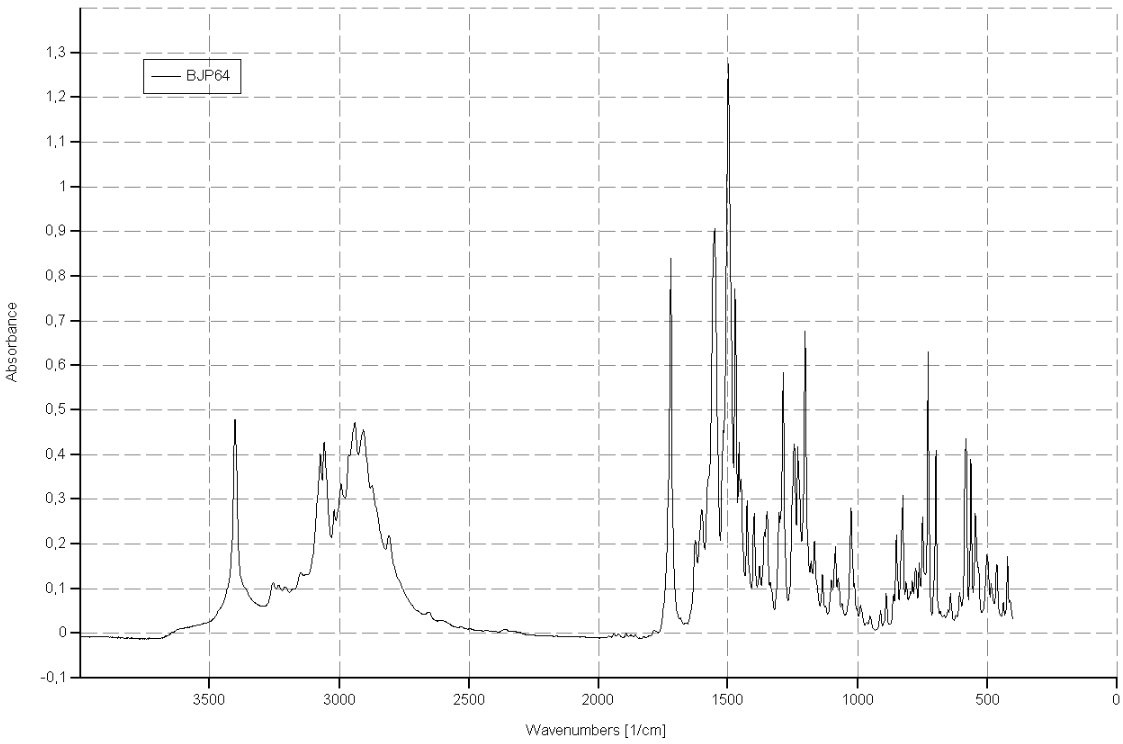

Figure S9. FTIR spectrum of **20b**

Ethyl 3-((1H-indol-3-yl)(phenyl)methyl)-7-chloro-4-oxo-1,4-dihydroquinoline-2-carboxylate (**20c**)

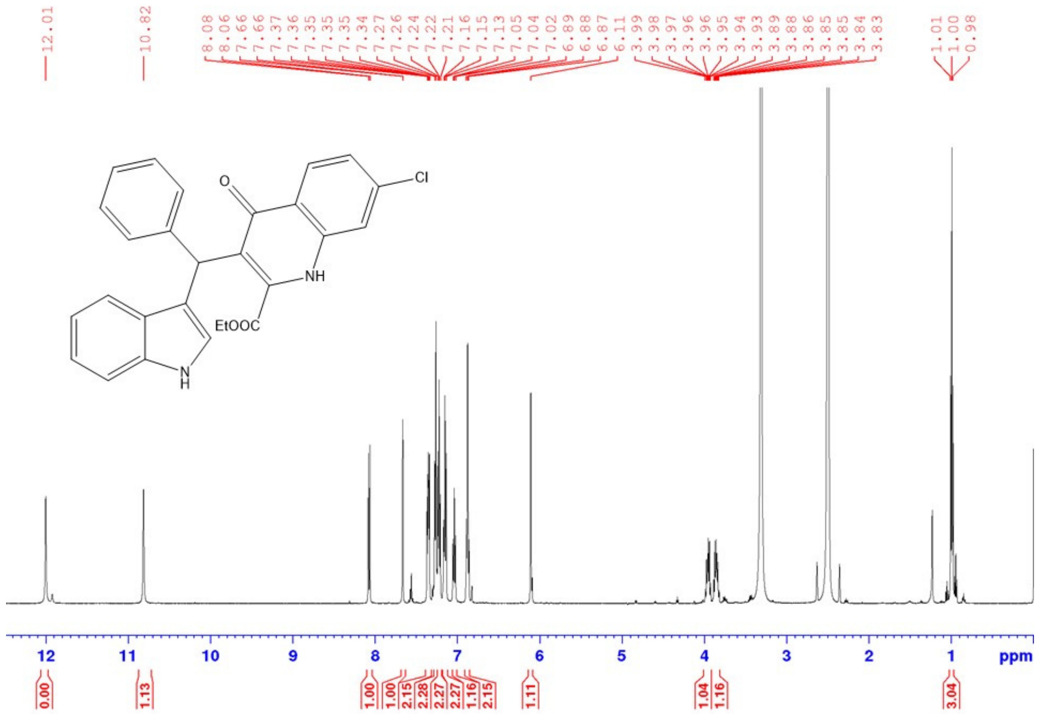

Figure S10. ^{1}H-NMR spectrum of **20c**

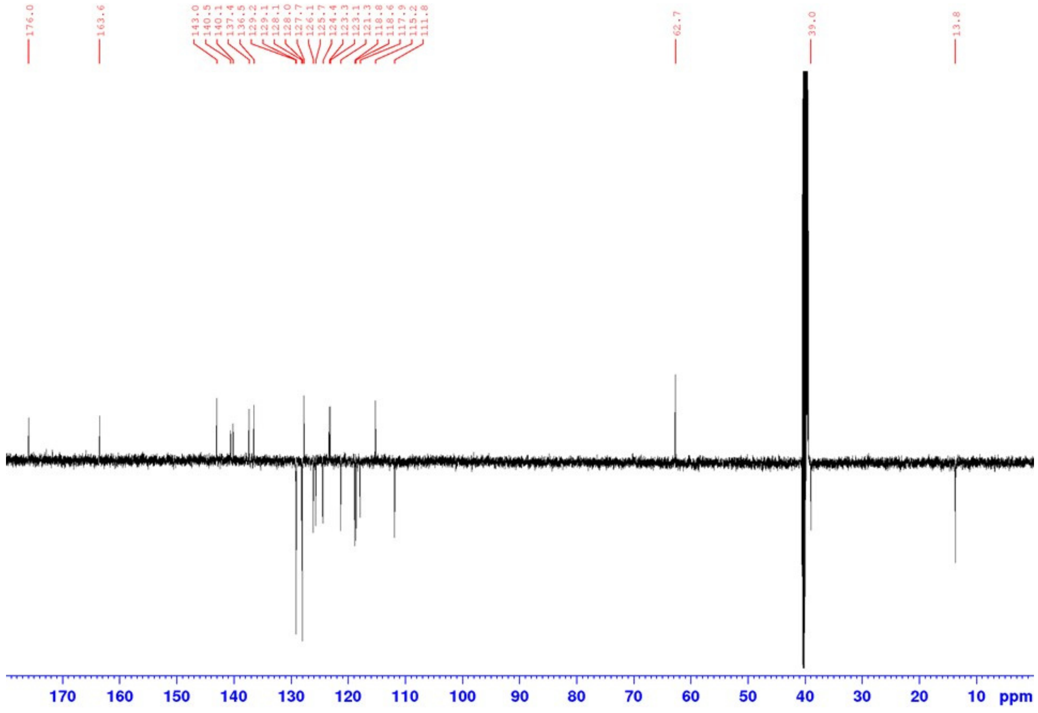

Figure S11. ^{13}C-NMR spectrum of **20c**

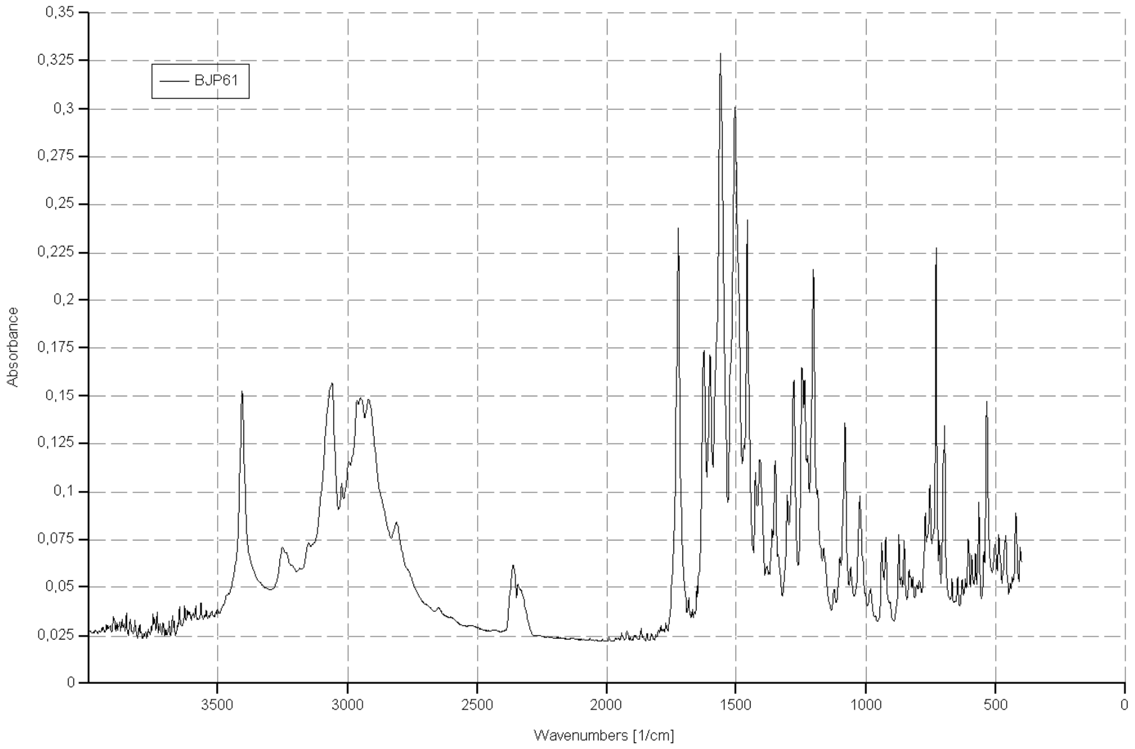

Figure S12. FTIR spectrum of **20c**

Ethyl 3-((1H-indol-3-yl)(phenyl)methyl)-8-chloro-4-oxo-1,4-dihydroquinoline-2-carboxylate (**20d**)

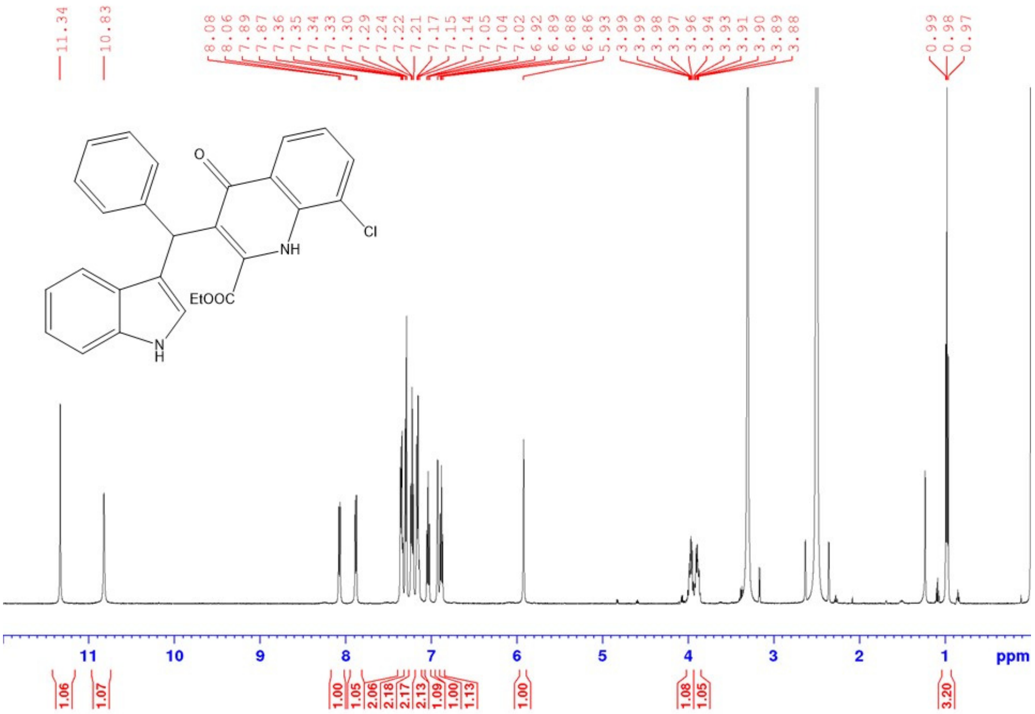

Figure S13. ^{1}H-NMR spectrum of **20d**

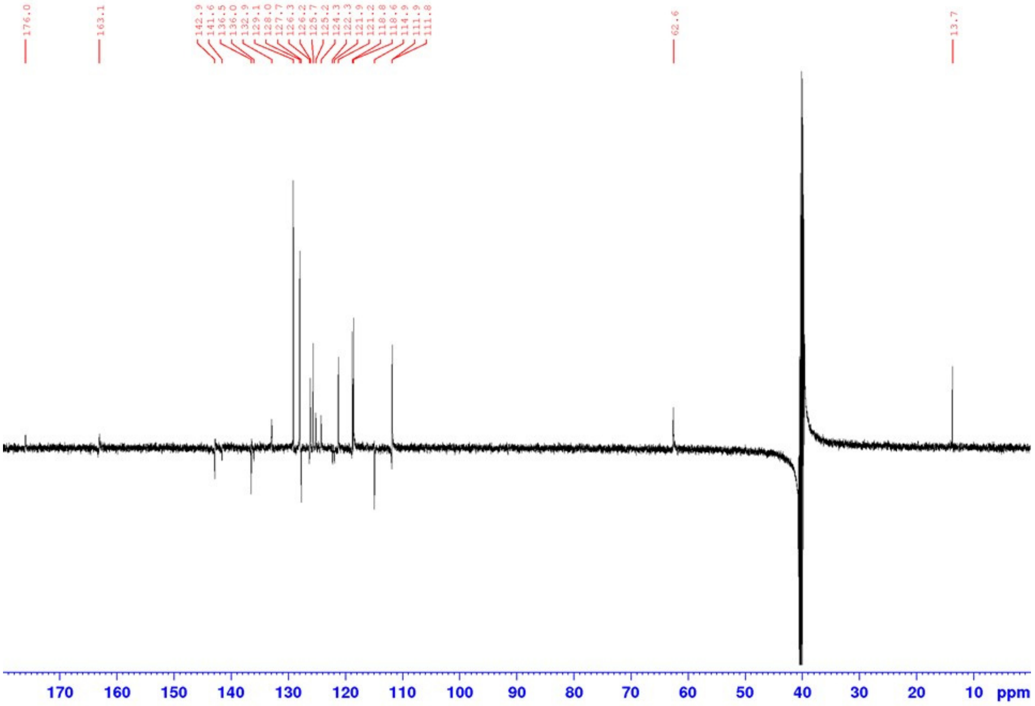

Figure S14. ^{13}C-NMR spectrum of **20d**

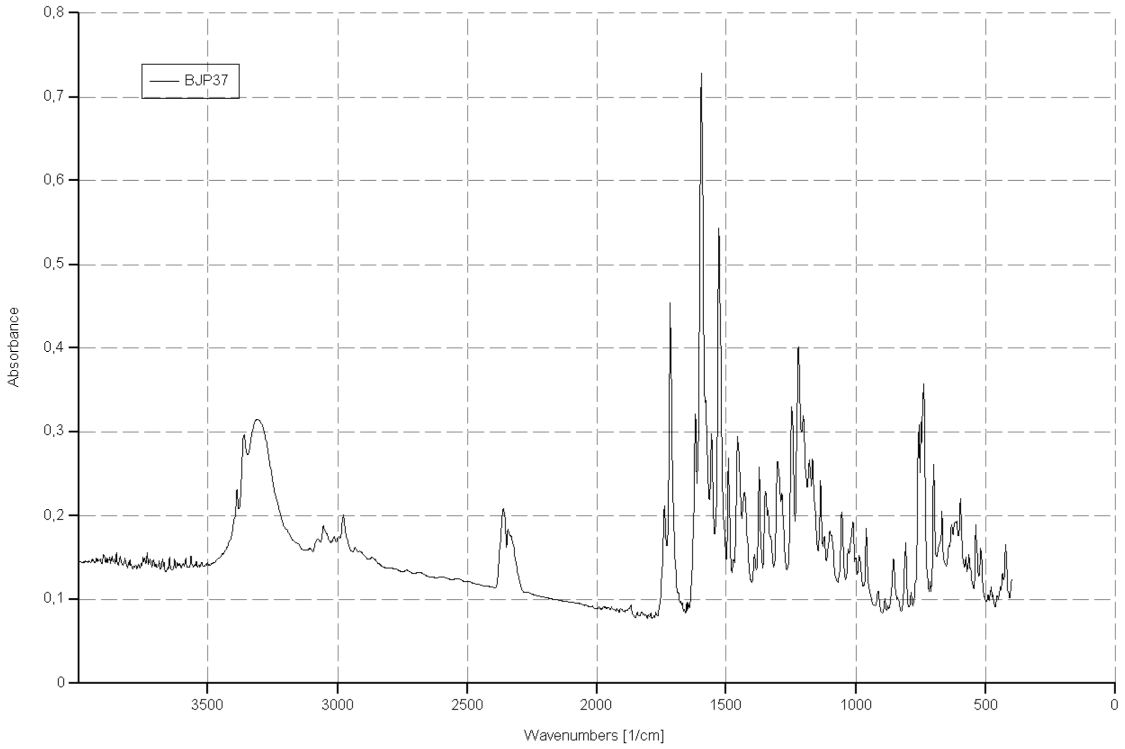

Figure S15. FTIR spectrum of **20d**

Ethyl 3-((1H-indol-3-yl)(phenyl)methyl)-5-methoxy-4-oxo-1,4-dihydroquinoline-2-carboxylate (**20e**)

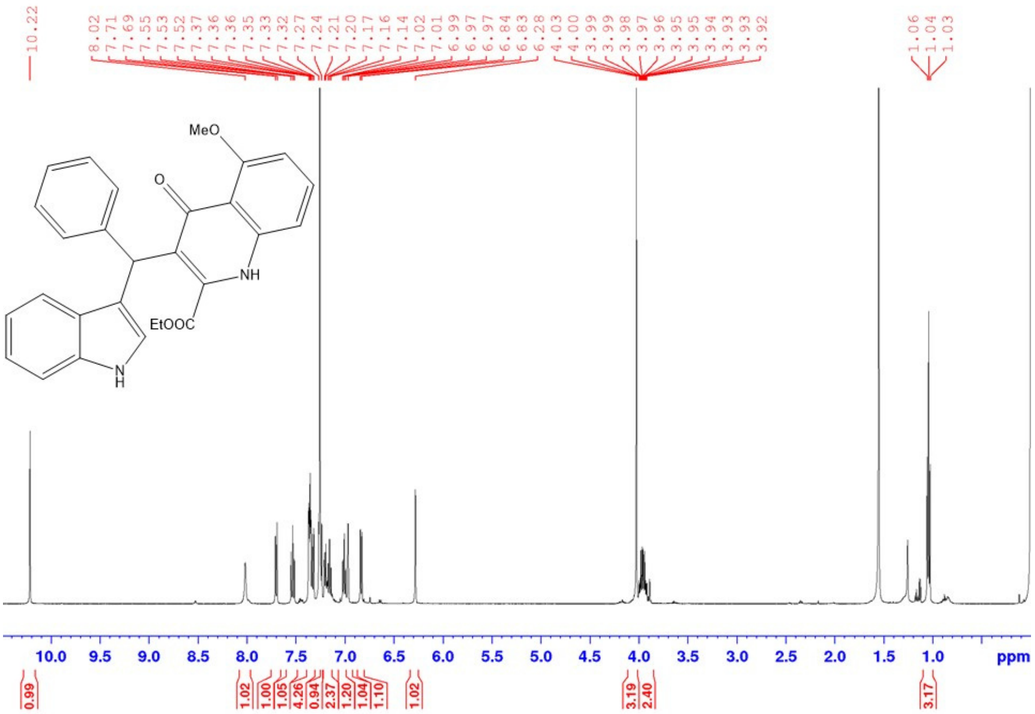

Figure S16. ^{1}H-NMR spectrum of **20e**

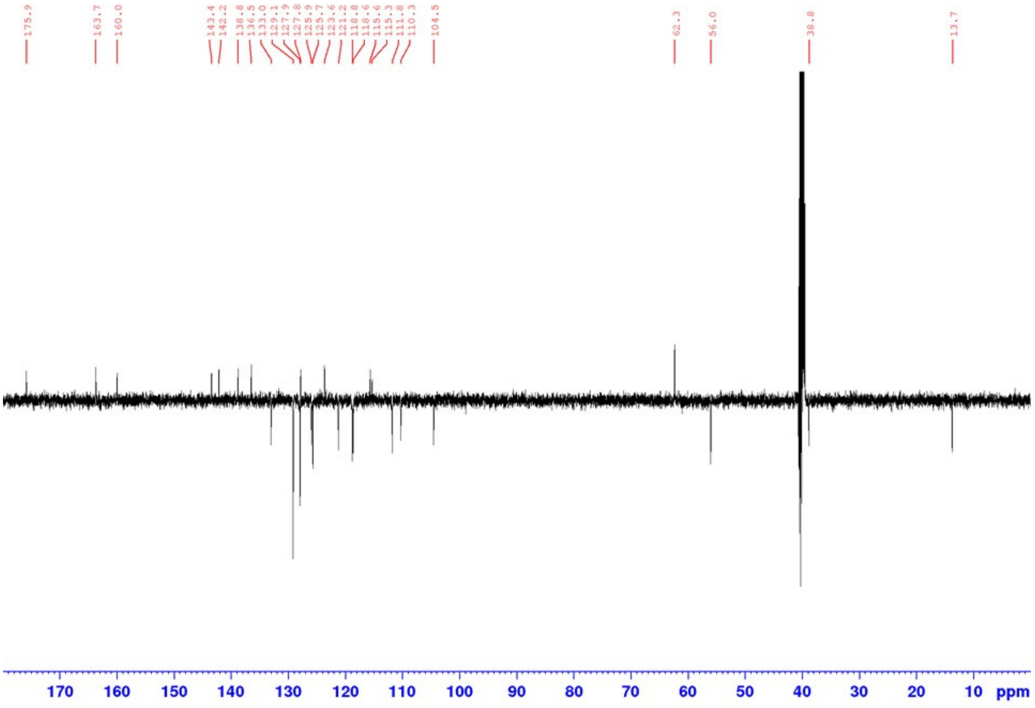

Figure S17. ^{13}C-NMR spectrum of **20e**

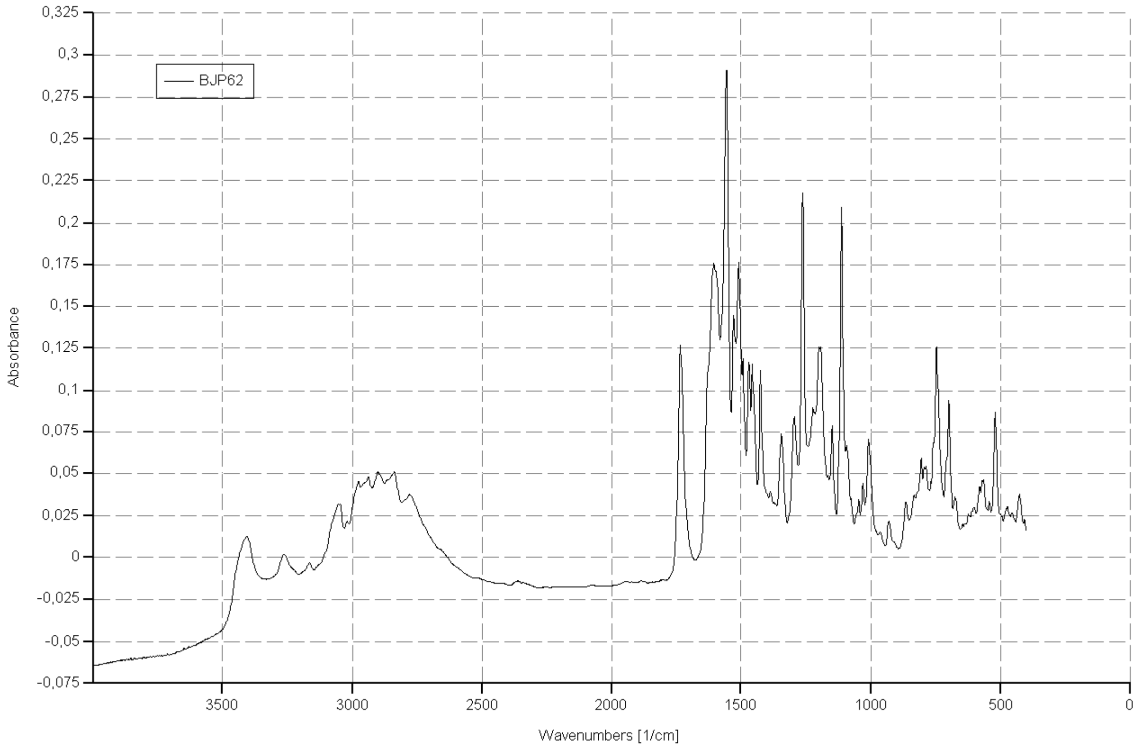

Figure S18. FTIR spectrum of **20e**

Ethyl 3-((1H-indol-3-yl)(phenyl)methyl)-6-methoxy-4-oxo-1,4-dihydroquinoline-2-carboxylate (**20f**)

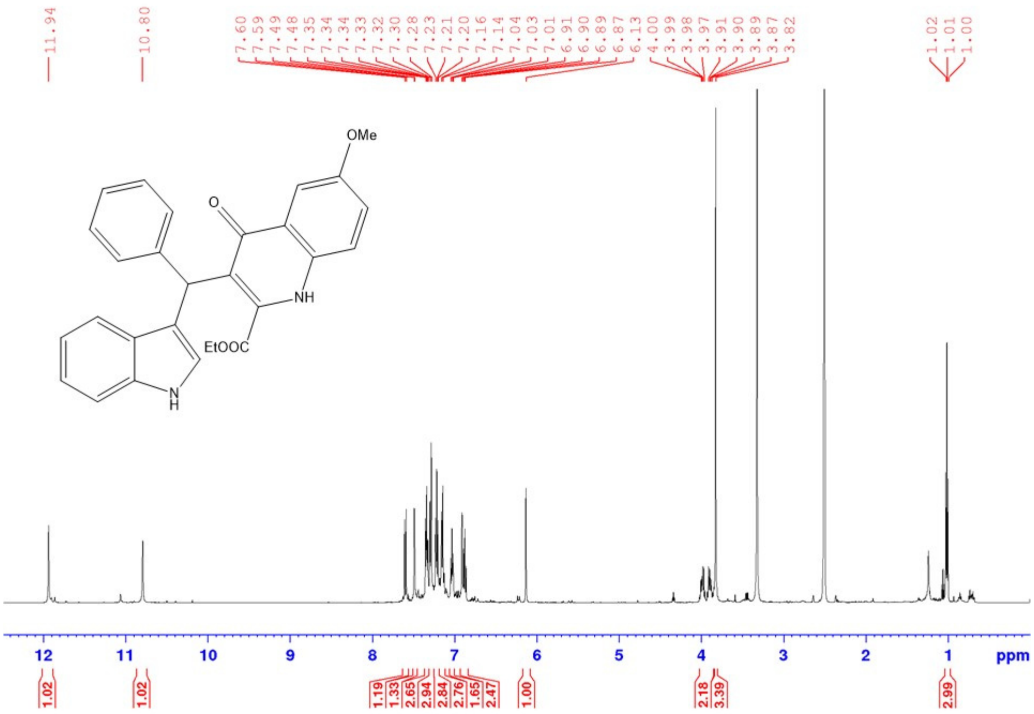

Figure S19. ^{1}H-NMR spectrum of **20f**

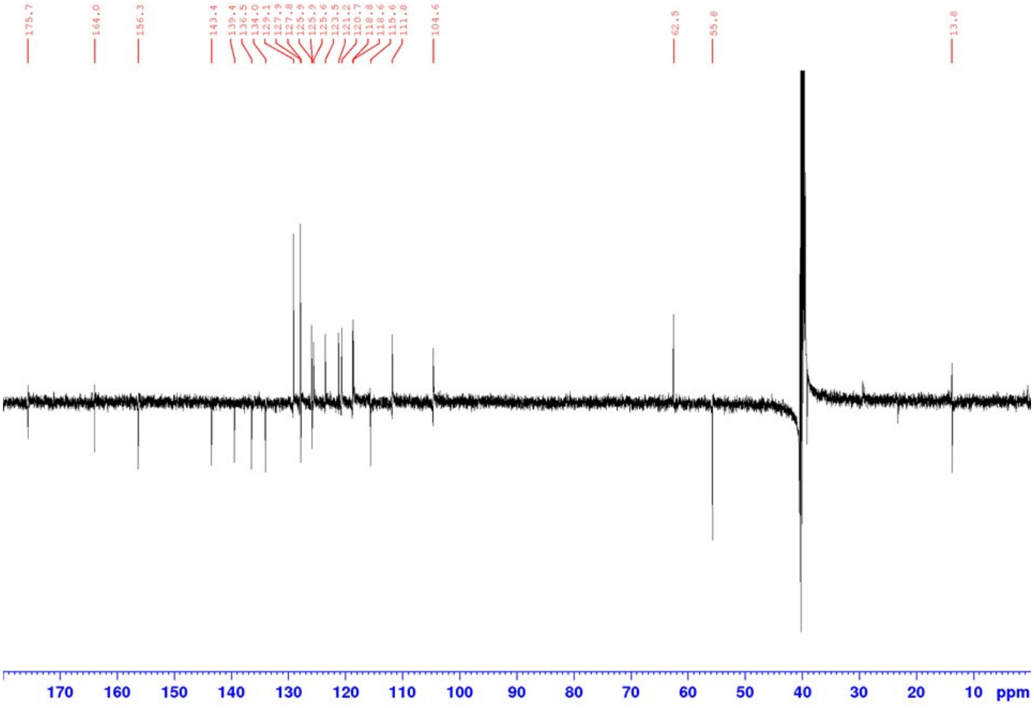

Figure S20. ^{13}C-NMR spectrum of **20f**

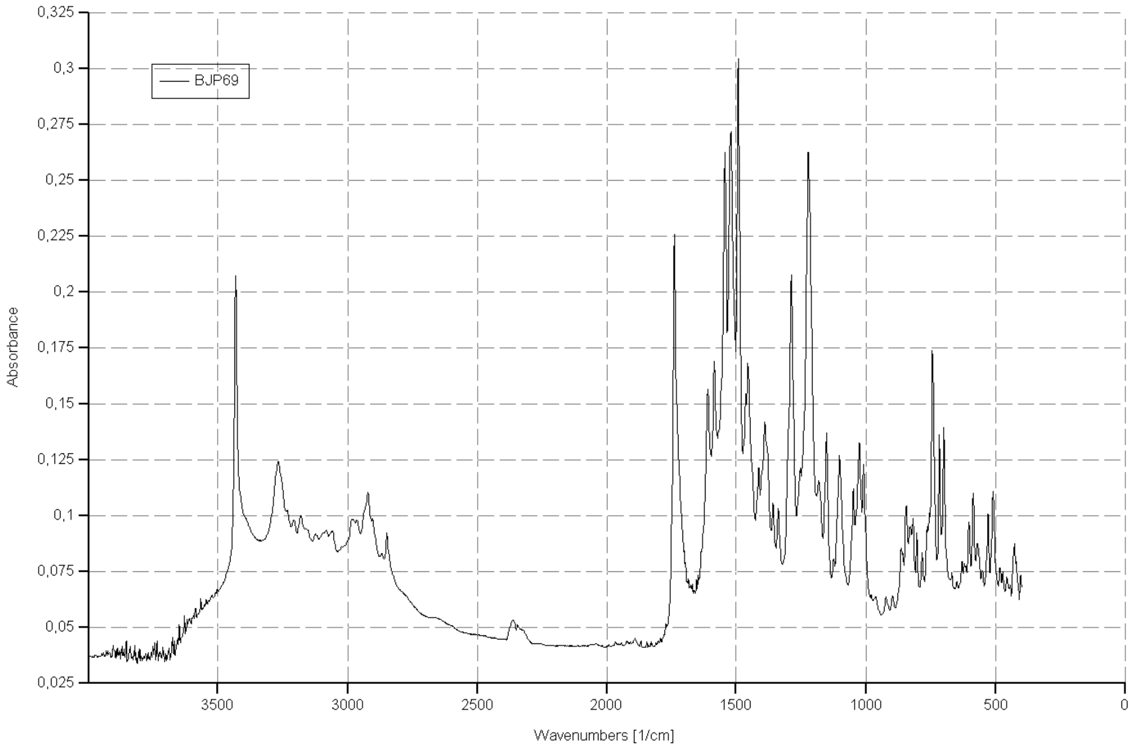

Figure S21. FTIR spectrum of **20f**

Ethyl 3-((1H-indol-3-yl)(phenyl)methyl)-7-methoxy-4-oxo-1,4-dihydroquinoline-2-carboxylate (**20g**)

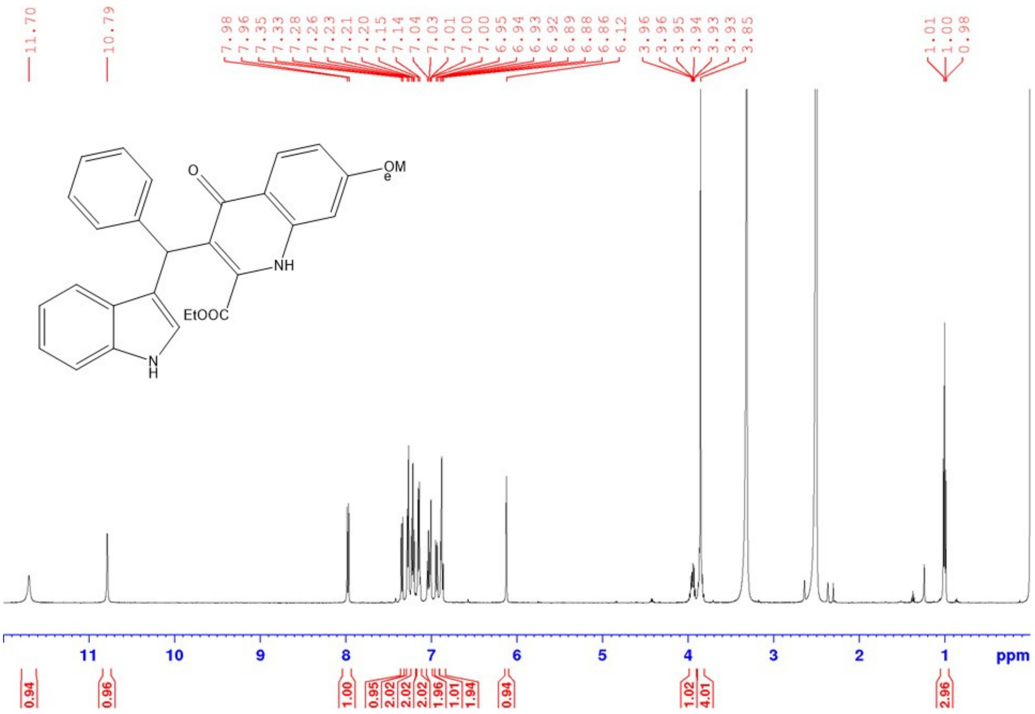

Figure S22. ^{1}H-NMR spectrum of **20g**

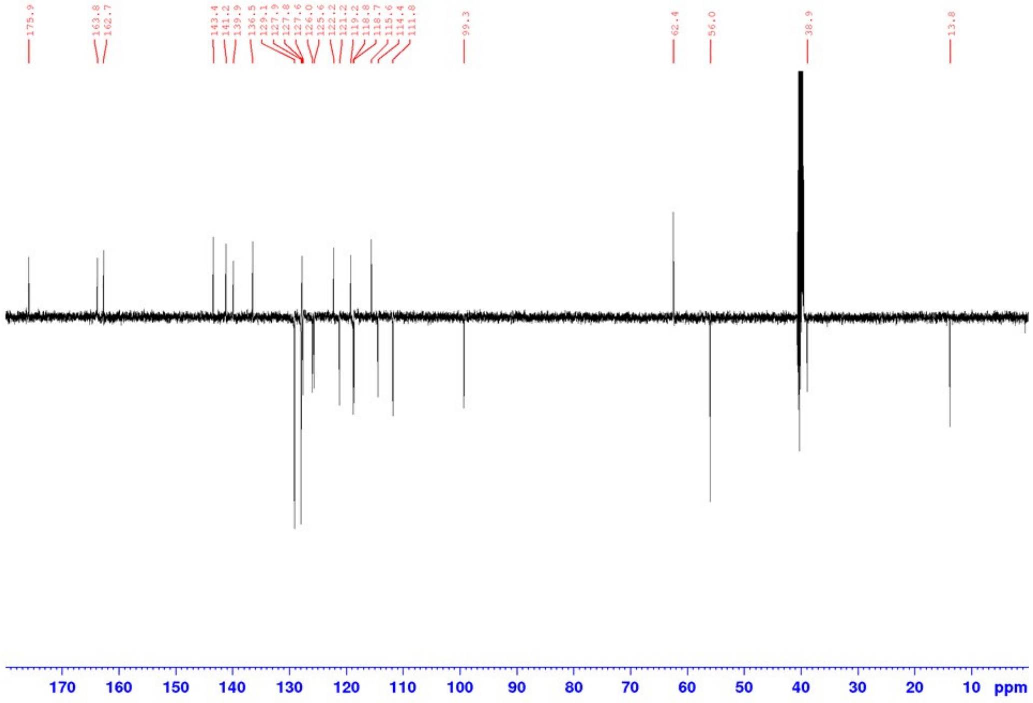

Figure S23. ^{13}C-NMR spectrum of **20g**

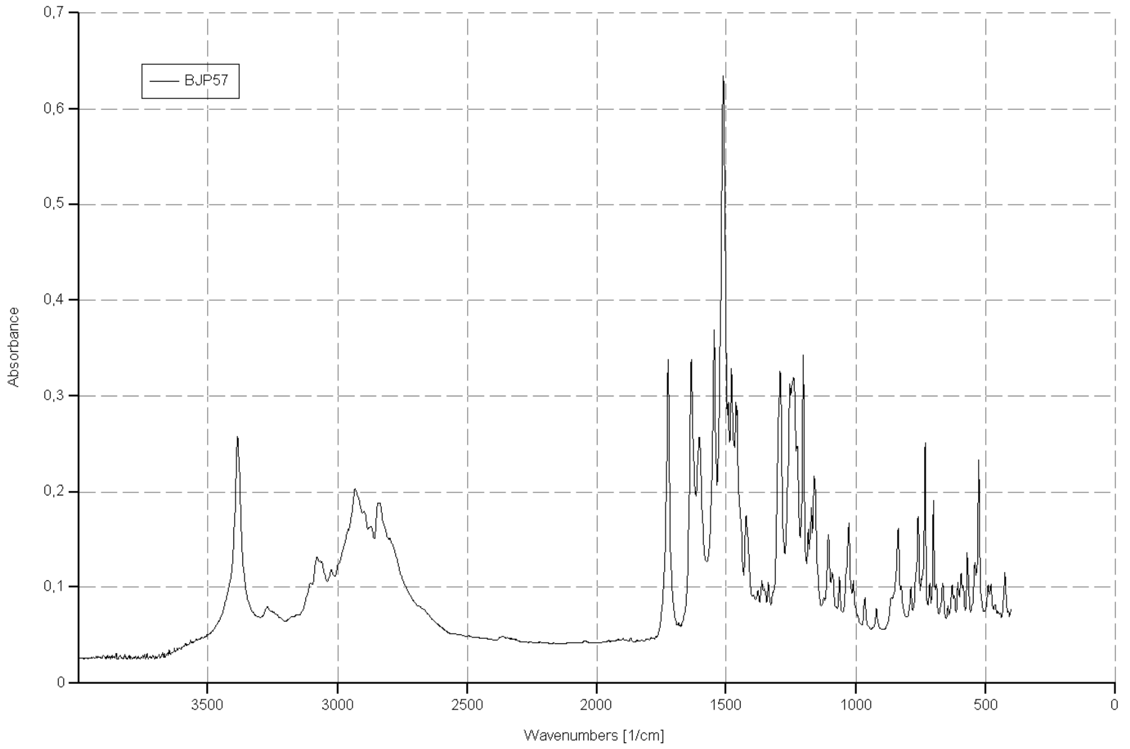

Figure S24. FTIR spectrum of **20g**

Ethyl 3-((1H-indol-3-yl)(phenyl)methyl)-8-methoxy-4-oxo-1,4-dihydroquinoline-2-carboxylate (**20h**)

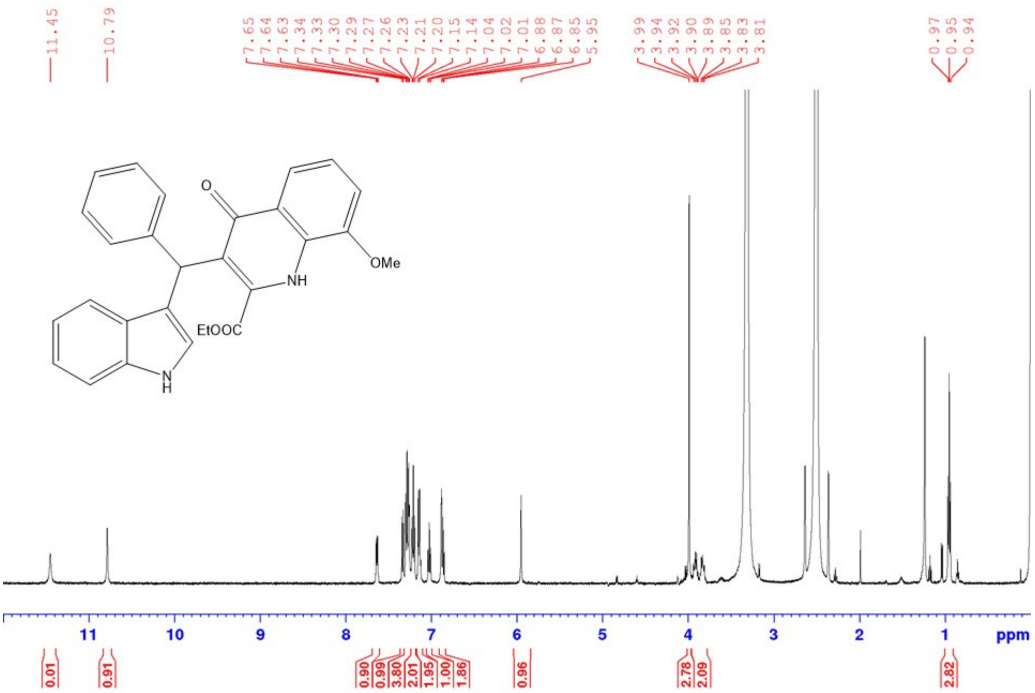

Figure S25. ^{1}H-NMR spectrum of **20h**

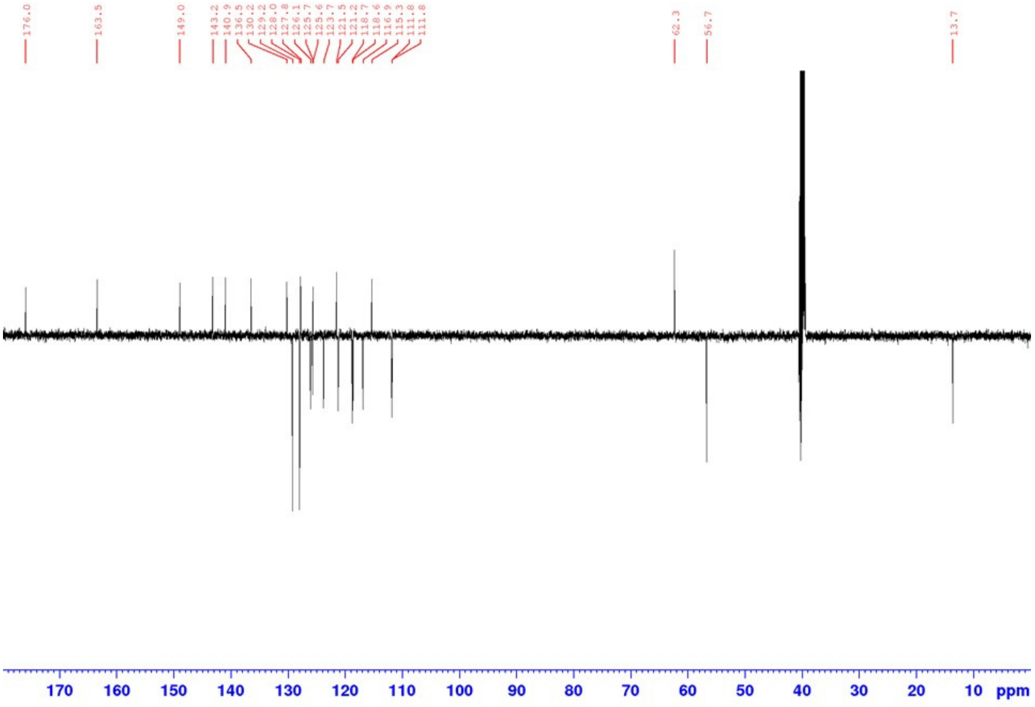

Figure S26. ^{13}C-NMR spectrum of **20h**

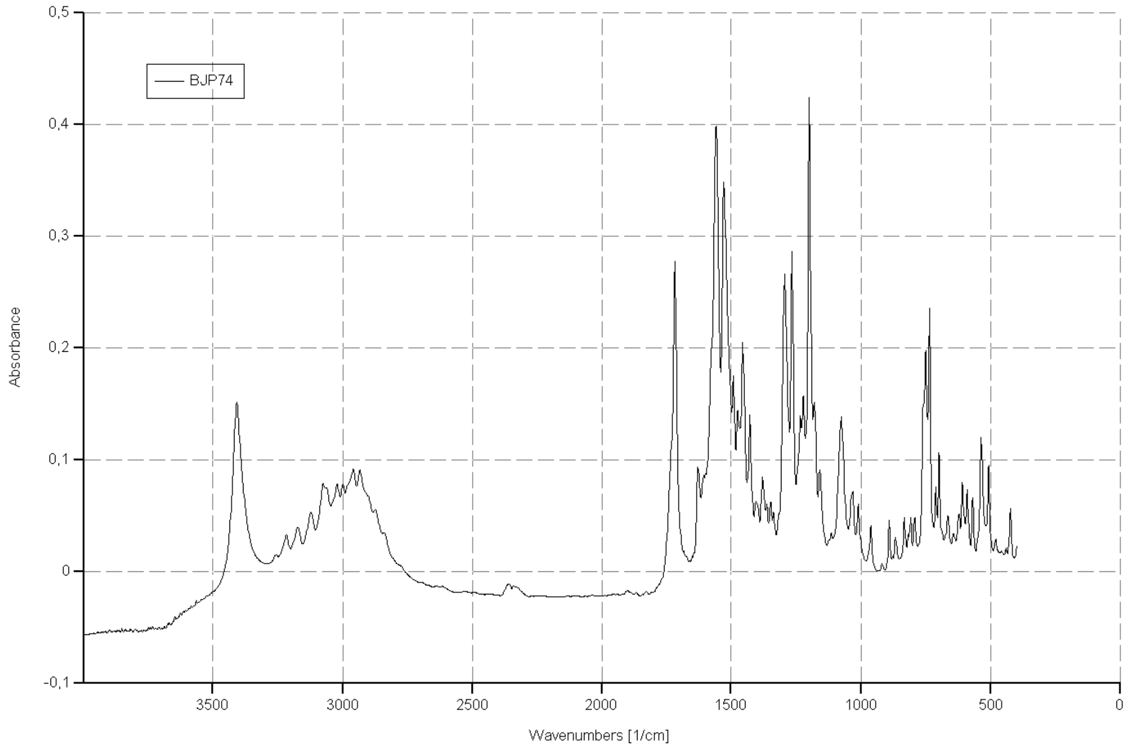

Figure S27. FTIR spectrum of **20h**

2-(((2-(Dimethylamino)ethyl)amino)(phenyl)methyl)naphthalen-1-ol (**16b**)

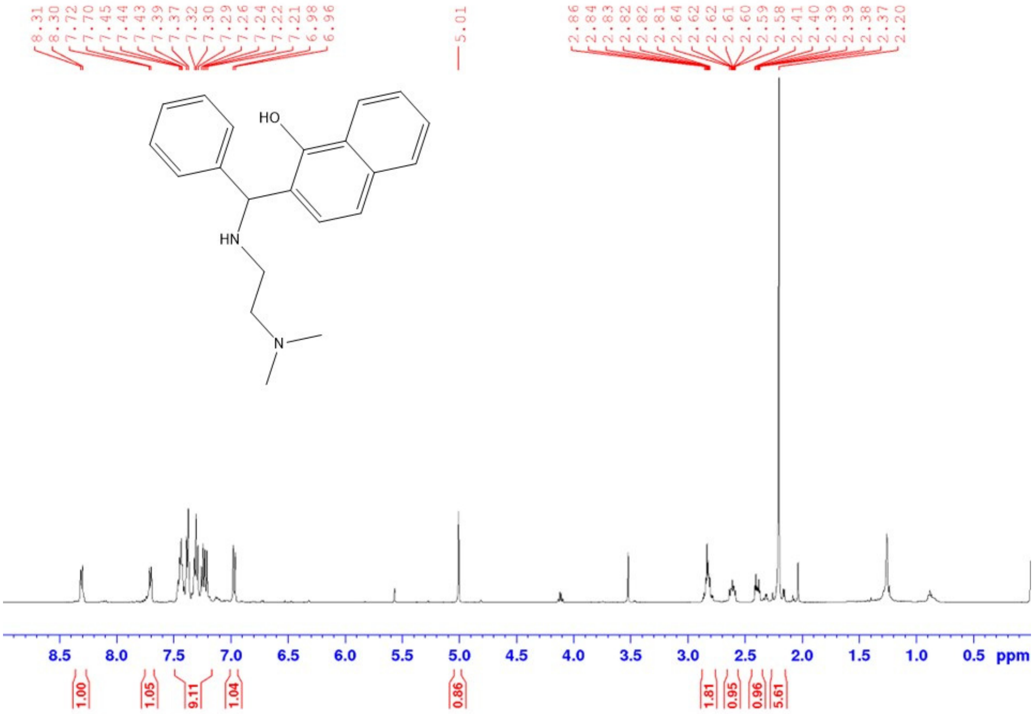

Figure S28. ^{1}H-NMR spectrum of **16b**

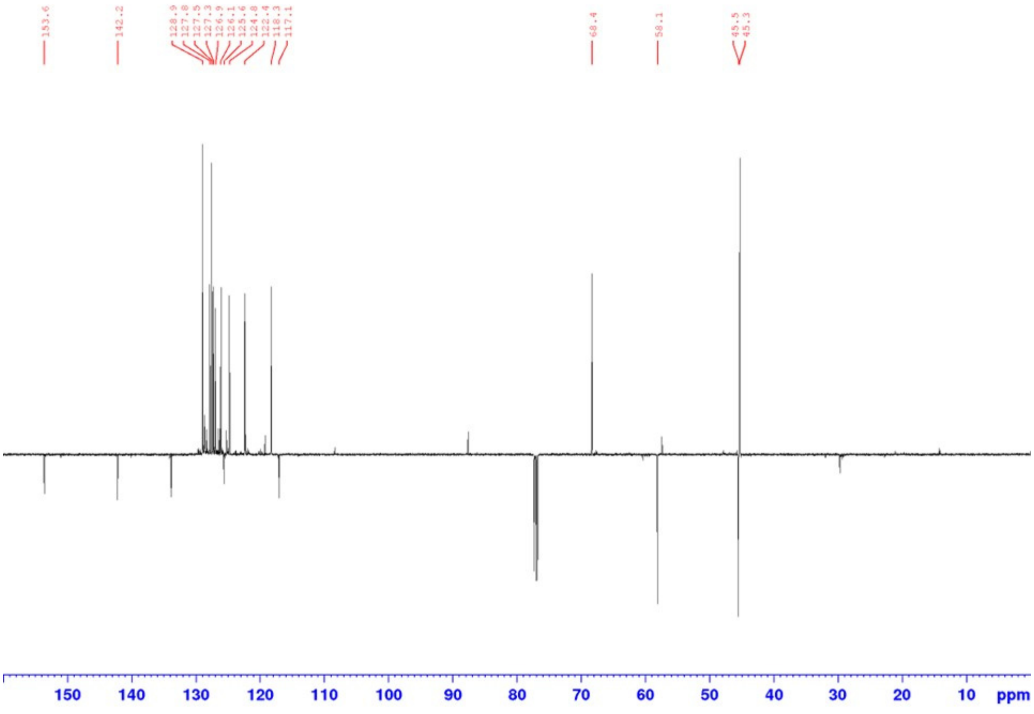

Figure S29. ^{13}C-NMR spectrum of **16b**

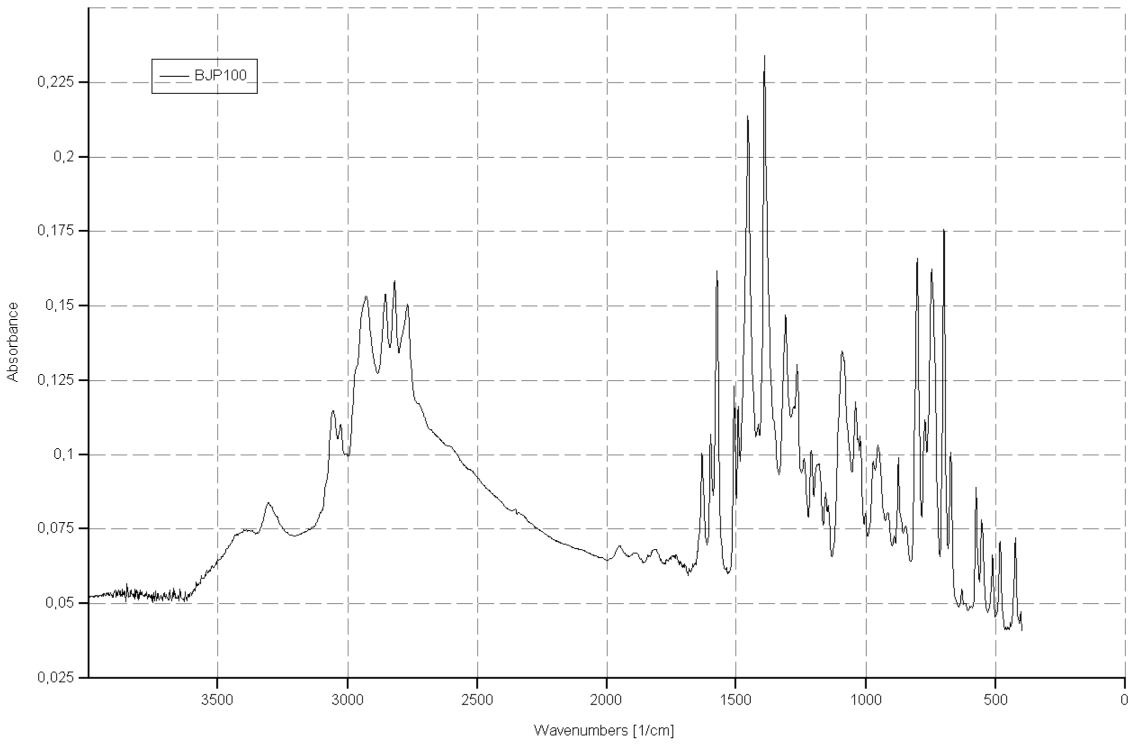

Figure S30. FTIR spectrum of **16b**

2-((1H-Indol-3-yl)(phenyl)methyl)naphthalen-1-ol (**18**)

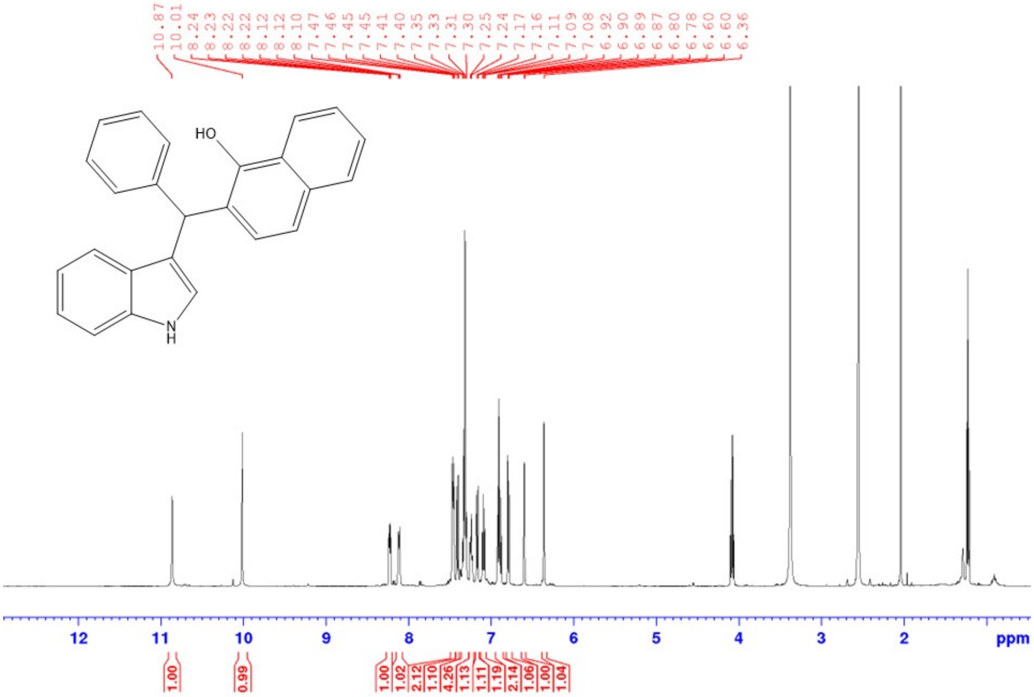

Figure S31. ^{1}H-NMR spectrum of **18**

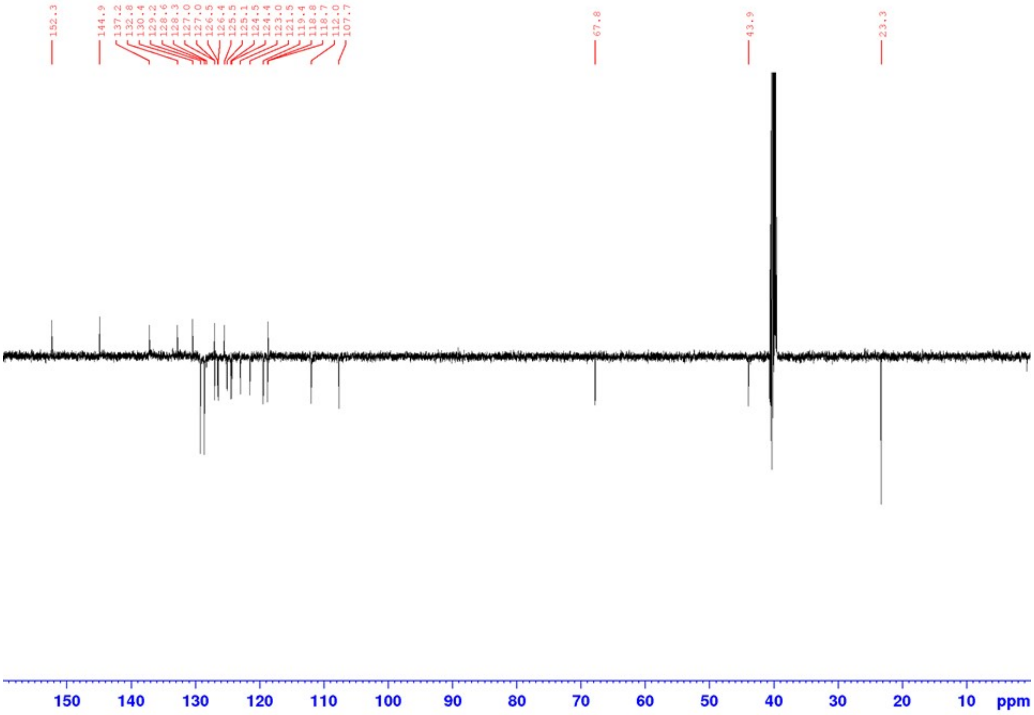

Figure S32. ^{13}C-NMR spectrum of **18**

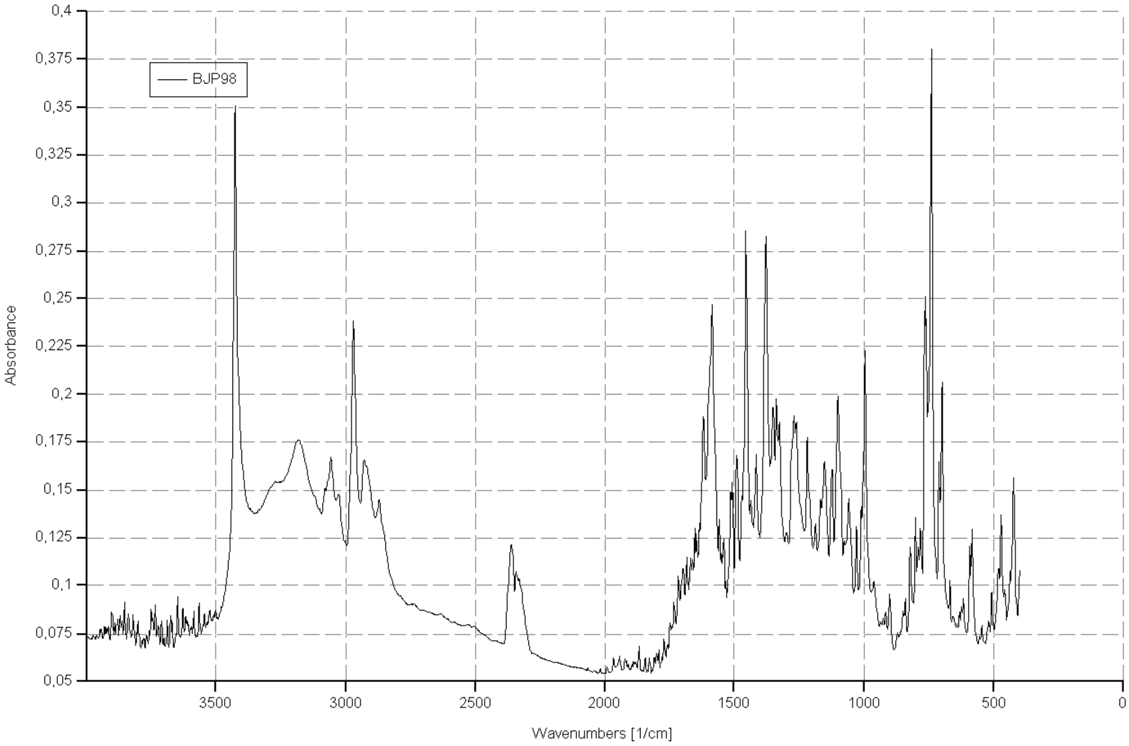

Figure S33. FTIR spectrum of **18**

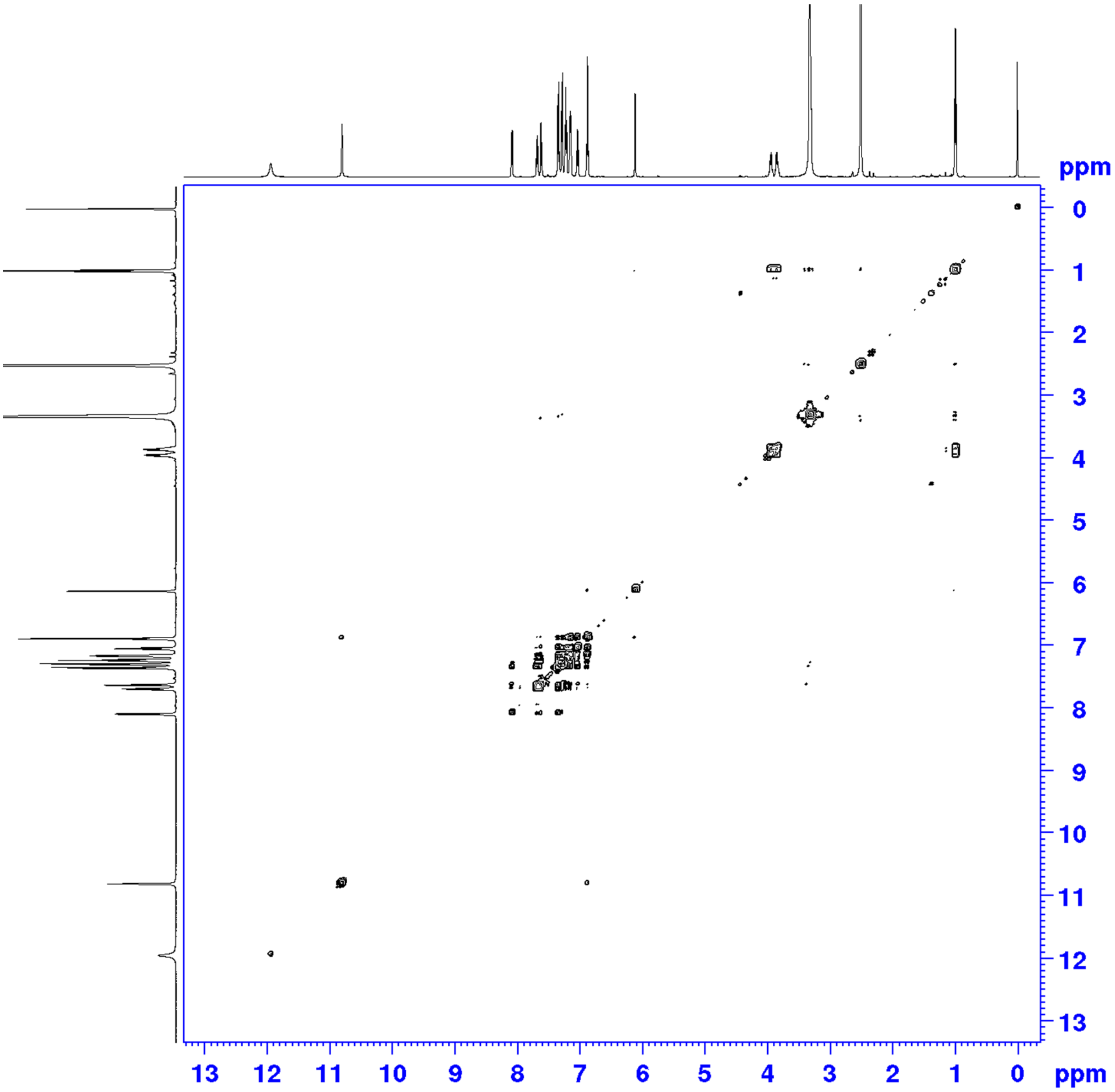

Figure S34. COSY spectrum of **7**

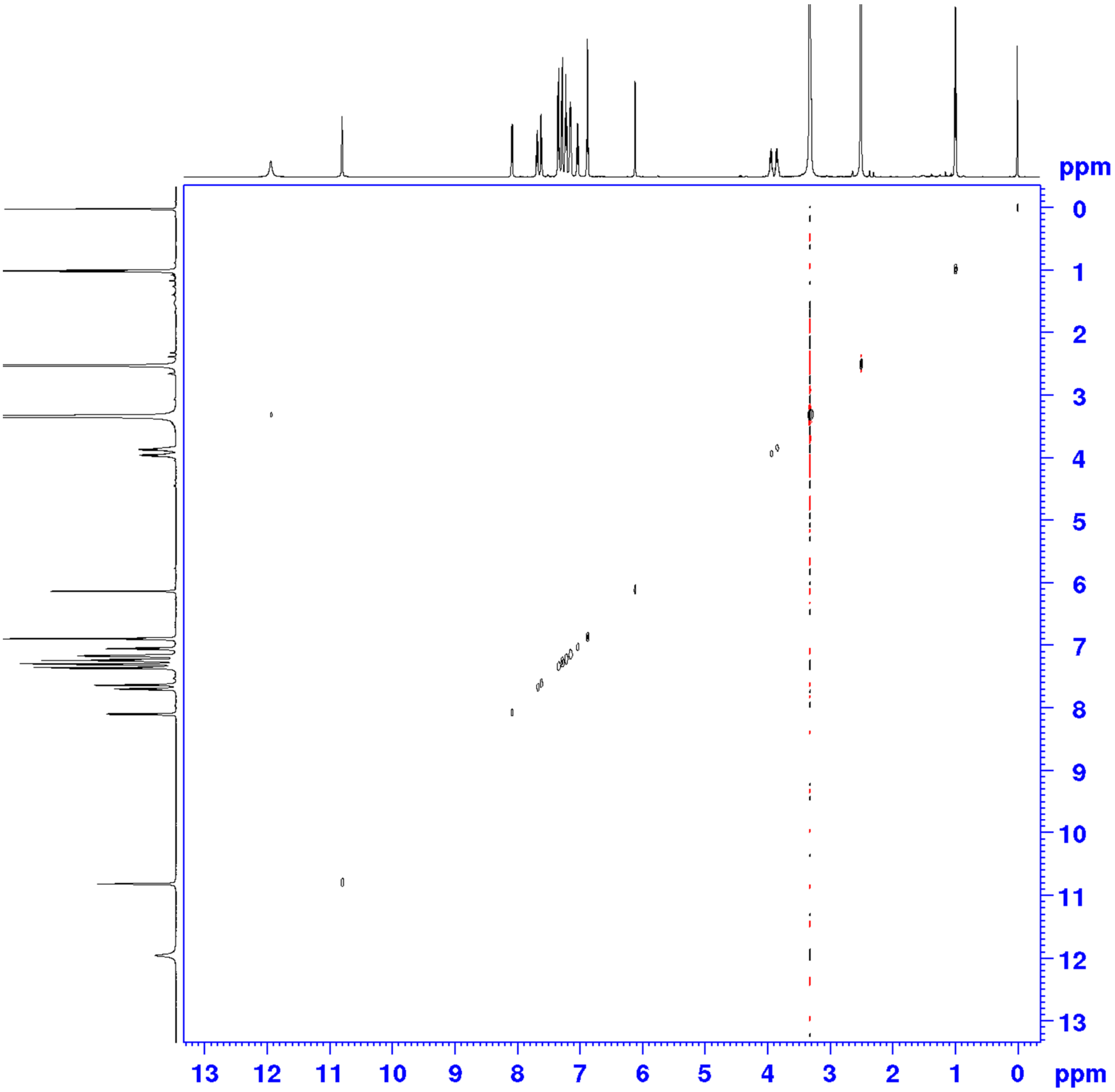

Figure S35. NOESY spectrum of **7**

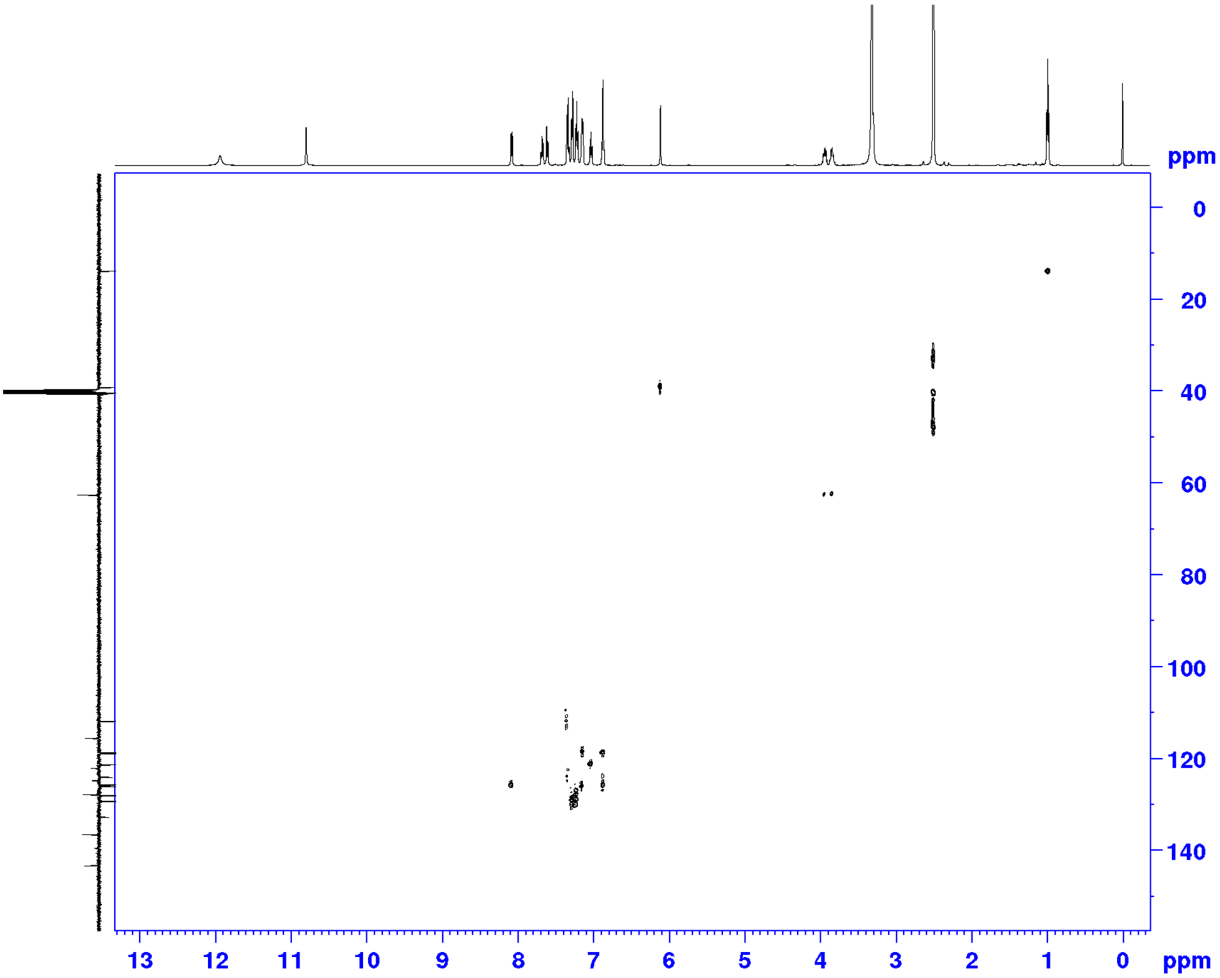

Figure S36. HSQC spectrum of **7**

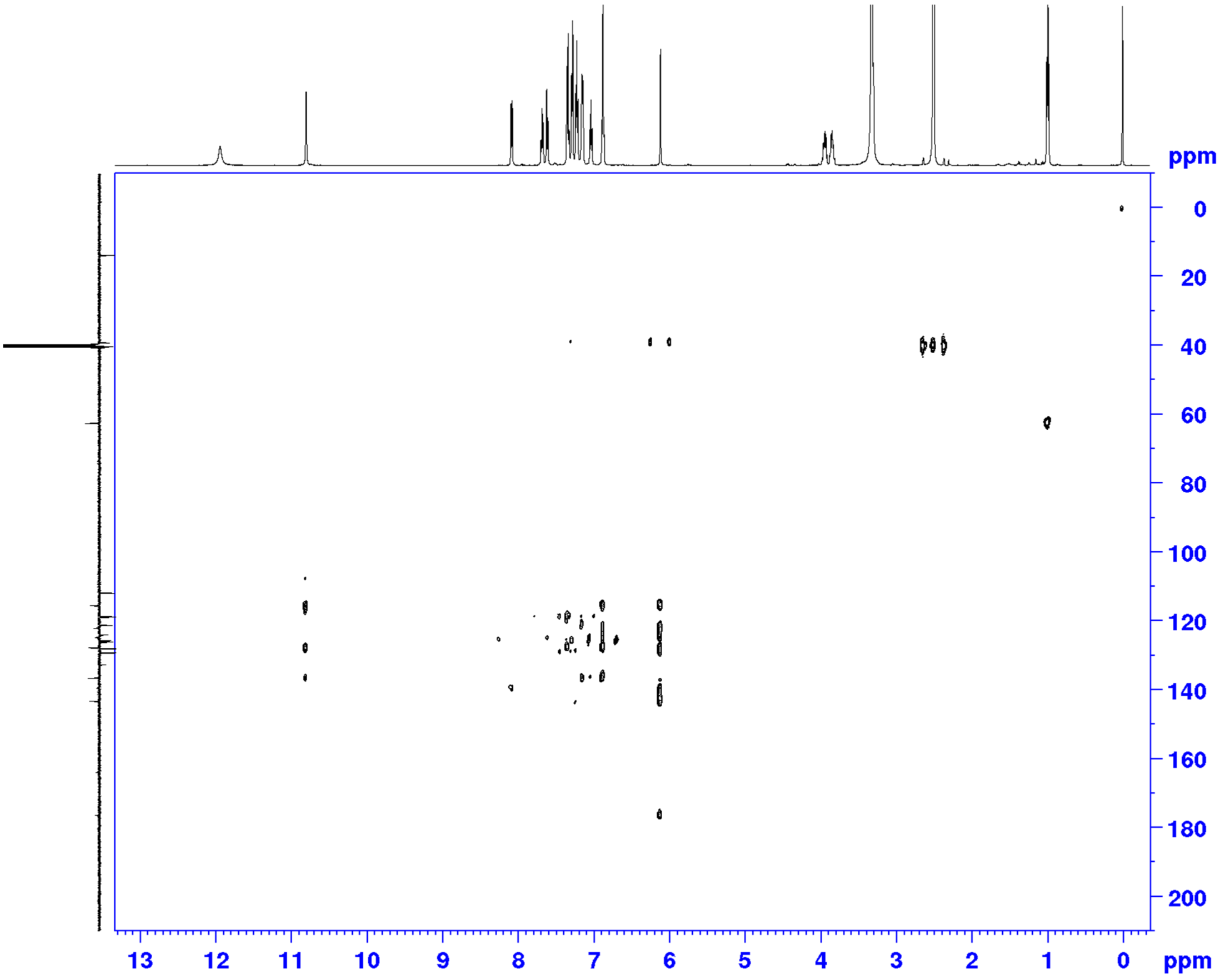

Figure S37. HMBC spectrum of **7**
